# BCKDH kinase promotes hepatic gluconeogenesis independent of BCKDHA

**DOI:** 10.1038/s41419-024-07071-0

**Published:** 2024-10-10

**Authors:** Feiye Zhou, Chunxiang Sheng, Xiaoqin Ma, Tianjiao Li, Xing Ming, Shushu Wang, Jialin Tan, Yulin Yang, Haipeng Sun, Jieli Lu, Jianmin Liu, Ruyuan Deng, Xiao Wang, Libin Zhou

**Affiliations:** 1 Department of Endocrine and Metabolic Diseases, Shanghai Institute of Endocrine and Metabolic Diseases, Ruijin Hospital, Shanghai Jiao Tong University School of Medicine, Shanghai, 200025 China; 2 Shanghai National Clinical Research Center for Metabolic Diseases, Key Laboratory for Endocrine and Metabolic Diseases of the National Health Commission of the PR China, Shanghai National Center for Translational Medicine, Ruijin Hospital, Shanghai Jiao Tong University School of Medicine, Shanghai, 200025 China; 3 Department of Endocrine and Metabolic Diseases, Renji Hospital, Shanghai Jiao Tong University School of Medicine, Shanghai, 200127 China; 4 NHC Key Laboratory of Hormones and Development, Tianjin Key Laboratory of Metabolic Diseases, Center for Cardiovascular Diseases, The Province and Ministry Co-sponsored Collaborative Innovation Center for Medical Epigenetics, Chu Hsien-I Memorial Hospital & Tianjin Institute of Endocrinology, Tianjin Medical University, Tianjin, 300134 China; 5 Department of Gastroenterology and Hepatology, Zhongshan Hospital, Fudan University, 200032, China; Shanghai Institute of Liver Disease, Shanghai, 200032 China

**Keywords:** Diabetes, Metabolic disorders

## Abstract

Elevated circulating branched-chain amino acids (BCAAs) are tightly linked to an increased risk in the development of type 2 diabetes mellitus. The rate limiting enzyme of BCAA catabolism branched-chain α-ketoacid dehydrogenase (BCKDH) is phosphorylated at E1α subunit (BCKDHA) by its kinase (BCKDK) and inactivated. Here, the liver-specific BCKDK or BCKDHA knockout mice displayed normal glucose tolerance and insulin sensitivity. However, knockout of BCKDK in the liver inhibited hepatic glucose production as well as the expression of key gluconeogenic enzymes. No abnormal gluconeogenesis was found in mice lacking hepatic BCKDHA. Consistent with the vivo results, BT2-mediated inhibition or genetic knockdown of BCKDK decreased hepatic glucose production and gluconeogenic gene expressions in primary mouse hepatocytes while BCKDK overexpression exhibited an opposite effect. Whereas, gluconeogenic gene expressions were not altered in BCKDHA-silenced hepatocytes. Mechanistically, BT2 treatment attenuated the interaction of cAMP response element binding protein (CREB) with CREB-binding protein and promoted FOXO1 protein degradation by increasing its ubiquitination. Our findings suggest that BCKDK regulates hepatic gluconeogenesis through CREB and FOXO1 signalings, independent of BCKDHA-mediated BCAA catabolism.

## Introduction

Type 2 diabetes mellitus (T2DM) is a metabolic disorder characterized by chronic hyperglycemia. Hepatic gluconeogenesis plays an important role in maintaining glucose homeostasis [1], and abnormally enhanced gluconeogenesis is one of the pathological features of T2DM [2, 3]. Thus, targeting hepatic gluconeogenesis has emerged as a promising strategy to develop more effective therapies for T2DM.

Branched-chain amino acids (BCAAs), including leucine, isoleucine, and valine are essential amino acids for mammals [4]. Since the 1960s, researches have noted that elevated circulating BCAA is tightly associated with obesity and insulin resistance [5–8]. With the advent of metabolomics technologies, BCAAs and their metabolites (e.g., acylcarnitines, 3-HIB) have shown the potential to be better biomarkers for diabetes [9–12]. Therefore, a number of groups tried to apply genetic manipulation, pharmacological intervention, or BCAA restricted diets to uncover the molecular mechanisms underlying BCAA catabolism-regulated insulin sensitivity [8, 13–17]. However, little is known about the role of BCAA catabolism in the regulation of gluconeogenesis.

BCAA catabolism is initiated through a reversible conversion of leucine, isoleucine, and valine into their corresponding α-keto acids (BCKAs) catalyzed by branched-chain aminotransferase (BCAT). Then BCKAs undergo irreversible oxidative decarboxylation mediated by branched-chain α-ketoacid dehydrogenase (BCKDH) enzyme complex to produce coenzyme A (CoA) esters [18], which are eventually oxidized in the tricarboxylic acid (TCA) cycle or incorporated into alternative pathways for gluconeogenesis, lipogenesis, ketone bodies or de novo cholesterol synthesis [15]. BCKDH kinase (BCKDK) phosphorylates the E1α subunit of BCKDH (BCKDHA) to inhibit its activity. Conversely, protein phosphatase PP2Cm (encoded by the gene PPM1K) removes this phosphorylation to promote BCKDHA activity [19, 20]. Though the role of BCAA metabolic enzymes in hepatic gluconeogenesis was rarely reported, the expression level of hepatic BCKDK in obese or diabetic rodents with enhanced gluconeogenesis was significantly increased [21–23]. Furthermore, insulin sensitivity was improved in these animals treated with BCKDK inhibitor 3,6-dichlorobenzo(b)thiophene-2-carboxylic acid (BT2) [8, 14]. However, whether BCKDK is involved in the regulation of hepatic gluconeogenesis remains unclear.

In the present study, we generated liver-specific BCKDK and BCKDHA knockout mice to determine their impacts on energy metabolism, especially on hepatic gluconeogenesis. Reduced hepatic glucose production and key gluconeogenic enzyme expressions were displayed in BCKDK-ablated mice, but not in BCKDHA-deleted mice. In vitro, pharmacological and genetic interventions of BCKDK in primary hepatocytes also revealed its involvement in the regulation of gluconeogenesis independent of BCKDHA. We further explored the mechanism underlying BCKDK-modulated hepatic gluconeogenesis.

## Material and methods

### Animals and diets

For the generation of liver-specific BCKDK knockout (BCKDK^Alb^ KO) mice, homozygous floxed BCKDK^loxP/loxP^ mice (loxP sites flanking exons 2–8) were crossed with transgenic mice expressing Cre-recombinase under the control of the albumin promoter (Alb-Cre). BCKDK floxed sites were confirmed by PCR analysis using forward and reverse primers: 5′-CCTCTCCATCTTCTTAATGCTGGG-3′ and 5′-GTCAGTAATAGGGGGATGGAGAGAT-3′, respectively. BCKDK^Alb^ KO (BCKDK^loxP/loxP^ Alb-Cre) mice were used for experiments, and their littermates (BCKDK^loxP/loxP^) were used as wild-type (WT) controls. 8-week-old BCKDK^Alb^ KO and WT mice were randomized to receive a normal chow diet (NCD, 10% of kcal fat) or high-fat diet (HFD, 60% of kcal fat) for 24 weeks. 4-week-old BCKDK^Alb^ KO and WT mice were fed 60% HFD supplemented with BCAA for 12 weeks. The BCAA mixture (L-leucine 10 g, L-isoleucine 5 g, and L-valine 5 g) was added into 1 L drinking water.

For the generation of liver-specific BCKDHA knockout (BCKDHA^Alb^ KO) mice, homozygous floxed BCKDHA^loxP/loxP^ mice (loxP sites flanking exons 4) were crossed with Alb-Cre mice. BCKDHA floxed sites were confirmed by PCR analysis using forward and reverse primers: 5′-GTGCCCAAGAACCAGCATGGAA-3′ and 5′-TCAACACAGCTTCTGCTTCCTGG-3′, respectively. Male BCKDHA^Alb^ KO (BCKDHA^loxP/loxP^ Alb-Cre) mice were used for experiments, and their littermates (BCKDHA^loxP/loxP^) were used as controls. 8-week-old BCKDHA^Alb^ KO and WT mice were fed 60% HFD for 16 weeks.

WT C57BL/6 and PP2Cm knockout male, age-matched mice were generated as previously reported [24]. Four-week-old male *db*/*db* mice and littermate lean *db*/m mice were purchased from Beijing Vital River Laboratory Animal Technology Co., Ltd. Male C57BL/6 mice for primary hepatocyte isolation were purchased from Shanghai Slack Experimental Center.

All the mice were on the C57BL/6 background and housed in a barrier facility with 12 h light/dark cycles and ad libitum access to water and food.

### Metabolic studies

Lean and fat mass were determined by EchoMRI (EchoMRI body composition analyzer) in unanesthetized mice. BCKDK^Alb^ KO mice were measured at 32 weeks old after feeding with HFD for 24 weeks and at 16 weeks old after feeding with HFD + BCAA for 12 weeks. NCD-fed BCKDHA^Alb^ KO mice were measured at 16 weeks old, and HFD-fed BCKDHA^Alb^ KO mice were measured at 24-week-old.

For glucose tolerance test (GTT), mice were intraperitoneally injected with 2 g/kg body weight of glucose after 16 h fasting (1 g/kg body weight of glucose for mice fed HFD or HFD + BCAA). For pyruvate tolerance test (PTT), mice were intraperitoneally injected with 2 g/kg body weight of pyruvate after 16 h fasting. For insulin tolerance test (ITT), mice were intraperitoneally injected with 0.75 U/kg body weight of insulin after 6 h fasting. An Oxymax Comprehensive Lab Animal Monitoring System (CLAMS) was used for measuring 24 h profiles of oxygen consumption (VO_2_) and carbon dioxide (VCO_2_), respiratory exchange ratio (RER), and locomotor activity.

### Primary hepatocyte isolation and culture

Primary mouse hepatocytes were isolated from C57BL/6 mice by a two-step perfusion technique as described [25]. Cells were treated with 100 μM 8-Bromoadenosine 3′,5′-cyclic monophosphate (8-Br-cAMP) (Sigma), 200 μM BT2 (MedChemExpress), or 10 μg/ml cycloheximide (CHX, Sigma).

### Adenovirus infection

Primary mouse hepatocytes were infected with adenovirus according to manufacturer instructions for 24–48 h. The recombinant adenoviruses were generated by GeneChem (Shanghai, China).

### Glucose production assay

Primary mouse hepatocytes were seeded into 24-well plates and pre-treated with 100 nM dexamethasone for 16 h. Then the medium was replaced with glucose production buffer consisting of glucose-free DMEM supplemented with 1 mM sodium pyruvate, 10 mM sodium lactate, and 0.25% BSA. After 24 h, the cell culture medium was collected for measuring glucose content by a colorimetric glucose assay kit (Applygen Company).

### Real-time quantitative PCR (RT-qPCR)

Total RNA was extracted from mouse tissues or primary hepatocytes using Trizol regent. To quantify the transcript abundance of genes of interest, RT-qPCR was performed with a SYBR Green Premix Ex Taq (Takara) in an Applied Biosystems 7300 Real-Time PCR machine (Applied Biosystems). The primer sequences used for RT-qPCR are shown in Supplementary Table 1.

### Western blotting and immunoprecipitation

Tissues or cells were homogenized in lysis buffer (Cell Signaling Technology). Blotted membrane was imaged with a LAS-4000 Super CCD Remote Control Science Imaging System (Fuji). Immunoprecipitation assays were performed by incubating protein lysates with indicated antibodies for 2 h and then with protein A/G-agarose beads (Santa Cruz) overnight at 4 °C. The immunoprecipitates were washed and eluted with SDS loading buffer. Then standard western blotting was followed.

### Ubiquitylation assay

For FOXO1 ubiquitylation analysis, HepG2 cells were transfected with plasmids of HA-ubiquitin and Flag-FOXO1 as indicated. Protease inhibitor MG132 and BT2 were added 4 h before harvest. Cells were collected and lysed in 1% SDS buffer. Immunoprecipitation of lysed proteins was performed by adding anti-Flag antibody, and ubiquitinated FOXO1 was detected by immunoblotting with anti-HA antibody.

### RNA sequencing

RNA was extracted from primary mouse hepatocytes treated with or without 200 μM BT2 for 8 h in the presence of 100 μM 8-Br-cAMP. 1–2 μg total RNA from each sample was used to prepare the sequencing library by KAPA Stranded RNA-Seq Library Prep Kit (Illumina, California, USA). Sequencing was performed on Illumina NovaSeq 6000 for 150 cycles. After quality control, raw sequencing data was pre-treated into trimmed data and further compared with *Mus musculus* genome by using Hisat2 software. The differentially expressed genes and transcripts (measured by fragments per kilobase of exon per million reads mapped value) were identified by setting a threshold at fold-change ≥1.5, *p* value < 0.05.

### Statistics

Data were expressed as mean ± SEM. Comparisons were performed using analysis ofvariance for multiple groups or the Student’s *t* test for two groups. Statistical significance was established at *P* < 0.05.

## Results

### Increased expression of hepatic BCKDK protein in diabetic or fasting mice

**Fig. 1:**
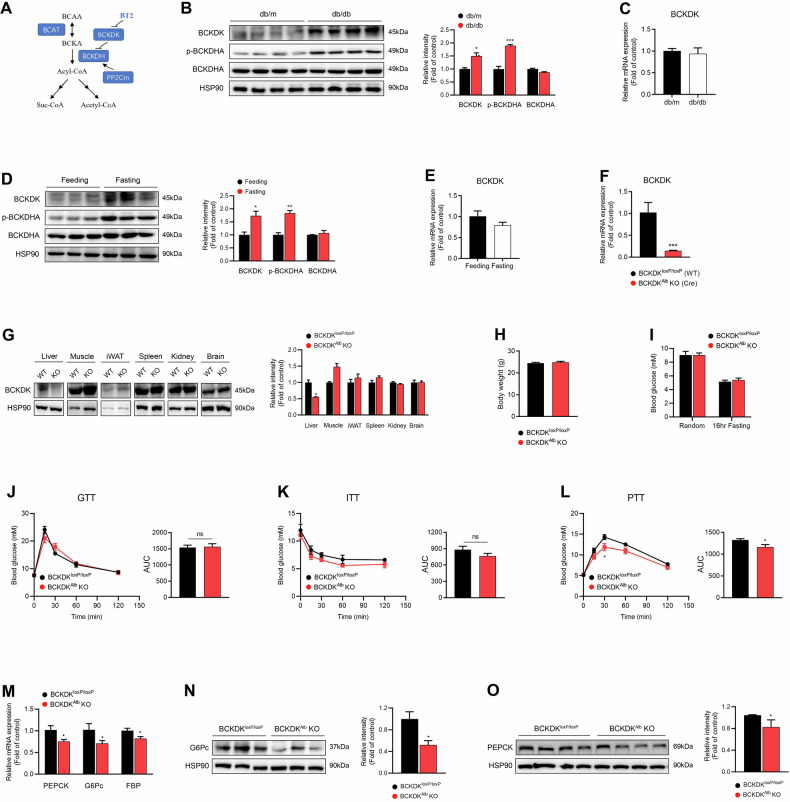
Liver-specific defect of BCKDK reduces hepatic glucose production in mice fed normal chow. **A** A schematic summary of BCAA catabolic pathway. **B** BCKDK protein expression and BCKDHA phosphorylation levels in the livers from *db*/m and *db*/*db* mice (*n* = 4). **C** BCKDK mRNA level in the livers from *db*/m and *db*/*db* mice (*n* = 4). **D** BCKDK protein expression and BCKDHA phosphorylation levels in the livers from C57BL/6 mice under feeding and fasting conditions (*n* = 3). **E** BCKDK mRNA expression in the livers from C57BL/6 mice underfeeding and fasting conditions (*n* = 3). **F** BCKDK mRNA expression in the liver was collected from BCKDK^Alb^ KO and WT mice (*n* = 3). **G** BCKDK protein expression in various tissues. **H** Body weight of 9-week-old male mice (*n* = 10). **I** Random and fasting blood glucose levels (*n* = 10). **J** Blood glucose levels during intraperitoneal glucose tolerance test (GTT) after 16 h fasting (*n* = 5). **K** Blood glucose levels during insulin tolerance test (ITT) after 6 h fasting (*n* = 5). **L** Blood glucose levels during intraperitoneal pyruvate tolerance test (PTT) after 16 h fasting (*n* = 8). **M** PEPCK, G6Pc, and FBP mRNA expressions in the liver (*n* = 10). **N**, **O** Western blot analysis of G6Pc and PEPCK expressions in the liver. Data are expressed as means ± SEM. ^*^P < 0.05, ^**^P < 0.01, ^***^P < 0.001 vs WT group.

Because of very low hepatic BCAT activity, the liver cooperates with skeletal muscle to achieve an appropriate BCAA shuttle toward oxidation [26]. A schematic summary of BCAA catabolic pathway was shown in Fig. 1A. Similar to the findings in obese mice and Zucker fatty rats [21–23], hepatic BCKDK protein expression and BCKDHA phosphorylation levels were obviously increased in *db*/*db* mice (Fig. 1B) compared with db/m mice in spite of no significant change of BCKDK mRNA level (Fig. 1C). In 8-week-old healthy C57BL/6 mice, BCKDK protein expression and BCKDHA phosphorylation in the liver were strongly induced after overnight fasting (Fig. 1D) while BCKDK mRNA expression remained unaltered (Fig. 1E). These findings suggest that hepatic BCKDK may be an important regulator of glucose homeostasis.

### Liver-specific defect of BCKDK reduces hepatic glucose production in mice fed normal chow

To investigate the metabolic effect of hepatic BCKDK in vivo, we used a Cre-loxP-mediated gene targeting strategy to selectively ablate BCKDK expression in the liver. BCKDK^loxP/loxP^ mice were crossed with Alb-Cre transgenic mice, generating BCKDK^Alb^ KO mice. BCKDK^loxP/loxP^ mice lacking Cre expression were used as control mice. RT-qPCR and Western blotting confirmed the obvious knockdown of BCKDK in the livers of BCKDK^Alb^ KO mice (Fig. 1F, G). Body weight as well as random and fasting blood glucose levels were comparable between two genotypes (Fig. 1H, I). Like mice with adeno-associated virus (AAV)-mediated shRNA knockdown of hepatic BDKDK in previous studies [16, 17], BCKDK^Alb^ KO mice exhibited similar glucose tolerance and insulin sensitivity compared to control mice (Fig. 1J, K). Interestingly, PTT revealed a significant reduction of hepatic glucose production in BCKDK^Alb^ KO mice (Fig. 1L), along with decreased hepatic mRNA and protein expressions of key gluconeogenic enzymes, including glucose-6-phosphatase (G6Pc), fructose 1,6-bisphosphatase (FBP), and phosphoenolpyruvate carboxykinase (PEPCK) (Fig. 1M–O). Taken together, liver-specific knockout of BCKDK indeed inhibits hepatic gluconeogenesis, but fails to improve glucose tolerance in mice fed with NCD.

### High-fat diet treatment abolishes hepatic BCKDK deletion-inhibited gluconeogenesis

To further determine whether the deletion of hepatic BCKDK confers beneficial effects in obese animals, BCKDK^Alb^ KO mice and WT littermates were challenged with HFD for 24 weeks. To our surprise, liver-specific knockout of BCKDK resulted in an exacerbation of HFD-induced body weight and fat mass gain, without affecting lean mass and liver weight (Fig. S1A–C). It was previously reported that BT2 treatment ameliorated insulin resistance in Zucker fatty rats and diet-induced obese (DIO) mice [14, 16]. Unlike BT2 administration, hepatic deletion of BCKDK dramatically elevated the glucose excursion during ITT (Fig. S1D), suggesting impaired insulin sensitivity in BCKDK^Alb^ KO mice under HFD conditions. Although NCD-fed BCKDK^Alb^ KO mice showed lower blood glucose levels in the process of PTT, this was not the case after 12 weeks of HFD feeding (Fig. S1E). Moreover, consumption of HFD for 24 weeks even led to a significant increment of hepatic glucose production in BCKDK^Alb^ KO mice compared to control mice (Fig. S1F). Additionally, VO_2_ and VCO_2_ levels were similar between the two genotypes (Fig. S1G, H). BCKDK^Alb^ KO mice showed a significant decrease in RER, signifying increased lipid utilization and reduced carbohydrate consumption (Fig. S1I). We also evaluated the general locomotor activity. Compared to control mice, the whole-body movement of BCKDK^Alb^ KO mice was significantly decreased during both light and dark periods (Fig. S1J). These data demonstrate that HFD treatment abolishes the inhibition of gluconeogenesis mediated by liver-specific BCKDK deletion, accompanied by more body weight gain and severe insulin resistance.

### Inhibitory effect of hepatic BCKDK knockout on gluconeogenesis under BCAA + HFD condition

**Fig. 2:**
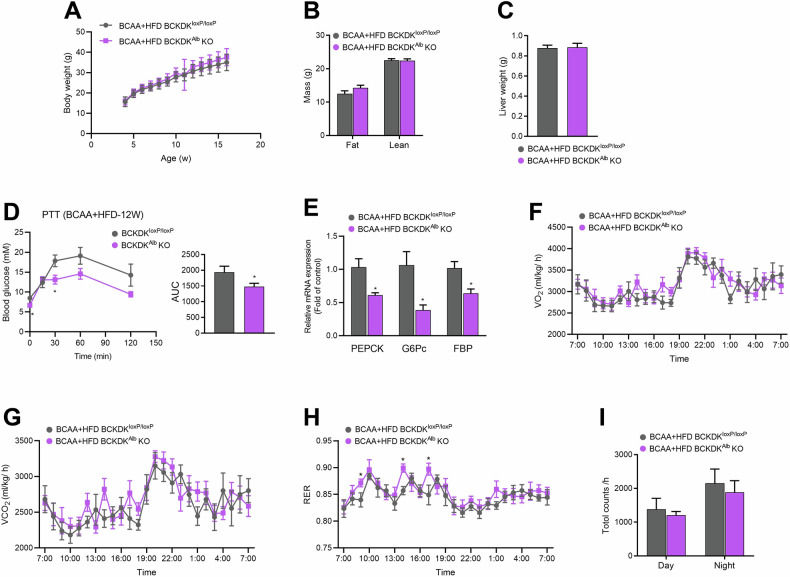
Inhibitory effect of hepatic BCKDK knockout on gluconeogenesis under BCAA + HFD condition. **A** Body weight of BCKDK^Alb^ KO and WT mice fed with BCAA + HFD throughout the study duration (*n* = 15). **B** Total fat mass and lean mass (*n* = 15). **C** Liver weight (*n* = 15). **D** Blood glucose levels during pyruvate tolerance test (PTT) after 12 weeks of BCAA + HFD feeding (*n* = 5–6). **E** PEPCK, G6Pc, and FBP mRNA expressions in the liver (*n* = 7). **F**–**H** Oxygen consumption (VO_2_), carbon dioxide (VCO_2_) production, and respiratory exchange ratios (RER) during 24 h (*n* = 8). **I** General locomotor activity during light and dark periods (*n* = 8). Data are expressed as means ± SEM. ^*^P < 0.05 vs WT group.

BCAA supplementation was reported to decrease the expression of G6Pc and PEPCK in the liver of rats fed with HFD [27]. Given that HFD treatment promoted hepatic glucose production in BCKDK^Alb^ KO mice, we further fed the mice with a BCAA + HFD diet, in which 60% of kcal fat was contained in food and a BCAA mixture was added into water. Body weight, fat mass, lean mass, and liver weight were comparable between BCKDK^Alb^ KO and WT mice after BCAA + HFD intervention for 12 weeks (Fig. 2A–C). However, BCKDK^Alb^ KO mice exhibited a remarkable improvement in hepatic glucose production compared with WT mice (Fig. 2D), with decreased hepatic expressions of gluconeogenic genes (Fig. 2E). Moreover, BCAA supplementation was sufficient to compensate for the reduced RER in HFD-fed BCKDK^Alb^ KO mice (Fig. 2F–H). No differences were detected in general locomotor activity between BCKDK^Alb^ KO and WT mice under BCAA + HFD condition (Fig. 2I).

### Liver-specific knockout of BCKDHA has no effect on gluconeogenesis in mice

**Fig. 3:**
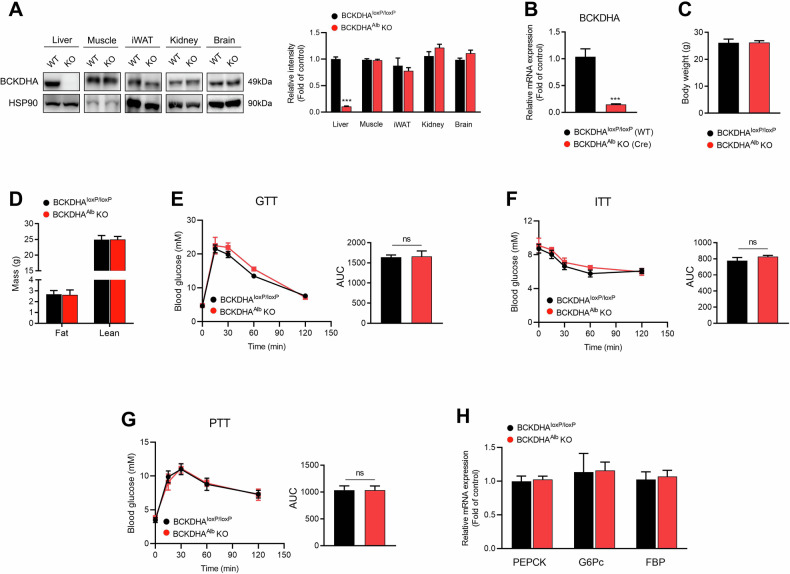
Metabolic effect of liver-specific knockout of BCKDHA on mice fed normal chow. **A** BCKDHA protein expression in various tissues. **B** BCKDHA mRNA expression in the liver collected from BCKDHA^Alb^ KO and WT mice (*n* = 3). **C** Body weight of 14-week-old male mice (*n* = 5). **D** Total fat mass and lean mass (*n* = 5). **E** Blood glucose levels during intraperitoneal glucose tolerance test (GTT) after 16 h fasting (*n* = 5). **F** Blood glucose levels during insulin tolerance test (ITT) after 6 h fasting (*n* = 5). **G** Blood glucose levels during intraperitoneal pyruvate tolerance test (PTT) after 16 h fasting (*n* = 4). **H** PEPCK, G6Pc, and FBP mRNA expressions in the liver (*n* = 5). Data are expressed as means ± SEM. ^***^P < 0.001 *vs* WT group.

To determine whether BCAA catabolism is involved in BCKDK-regulated hepatic gluconeogenesis, we crossed BCKDHA^loxP/loxP^ mice with Alb-Cre transgenic mice, generating BCKDHA^Alb^ KO mice (Fig. 3A, B). Compared with WT littermates (BCKDHA^loxP/loxP^ mice), no changes in body weight, fat mass, and lean mass were observed in BCKDHA^Alb^ KO mice (Fig. 3C, D). Like BCKDK^Alb^ KO mice, BCKDHA^Alb^ KO mice displayed normal glucose tolerance and insulin sensitivity compared with control mice (Fig. 3E, F). However, there were no differences in blood glucose levels during the whole process of PTT between the two genotypes (Fig. 3G), along with comparable mRNA expression levels of key gluconeogenic enzymes (Fig. 3H).

We further fed BCKDHA^Alb^ KO mice with HFD for 16 weeks. The body weight, fat mass, and lean mass of HFD-fed BCKDHA^Alb^ KO mice were similar to those of control mice (Fig. S2A, B). Additionally, HFD-fed BCKDHA^Alb^ KO mice also displayed similar glucose tolerance and pyruvate tolerance compared to control mice (Fig. S2C, D). No significant differences were detected in VO_2_, VCO_2_, RER, and general locomotor activity between the two genotypes (Fig. S2E–H). Overall, BCKDHA^Alb^ KO mice fail to exhibit abnormal hepatic glucose production opposite to those found in BCKDK^Alb^ KO mice, indicating that BCAA catabolism may not be involved in the regulation of hepatic gluconeogenesis mediated by BCKDK.

### BT2 inhibits gluconeogenesis in primary mouse hepatocytes

**Fig. 4:**
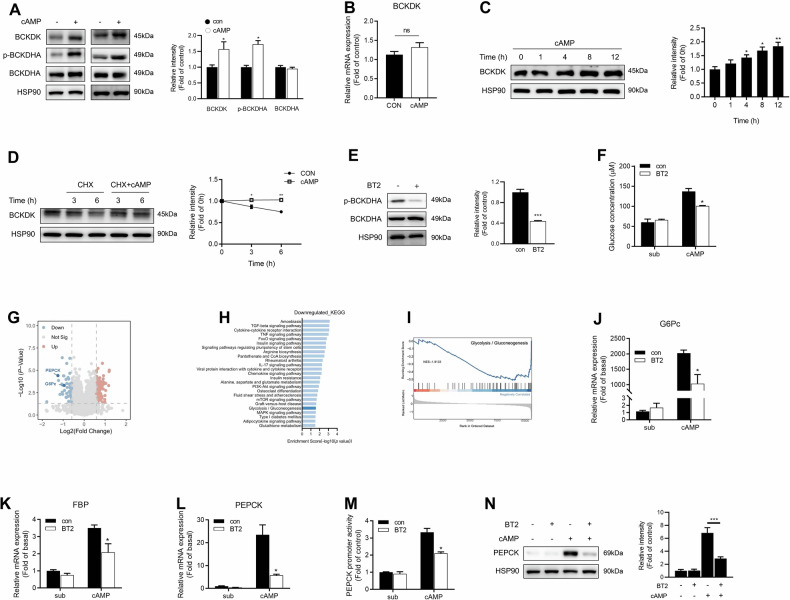
BT2 inhibits gluconeogenesis in primary mouse hepatocytes. **A** BCKDK protein expression and BCKDHA phosphorylation levels in primary mouse hepatocytes treated with 100 μM 8-Br-cAMP for 16 h. **B** BCKDK mRNA expression in primary mouse hepatocytes treated with 100 μM 8-Br-cAMP for 16 h. **C** Protein expression of BCKDK in primary mouse hepatocytes incubated with 100 μM 8-Br-cAMP for the indicated time. **D** BCKDK protein expression in primary mouse hepatocytes treated with 100 μM 8-Br-cAMP and 10 μg/ml cycloheximide (CHX) for the indicated time. Signal intensity was quantified by image J software for statistical comparison. **E** Phosphorylation level of BCKDHA in primary mouse hepatocytes incubated with 200 μM BT2 for 1 h. **F** Primary mouse hepatocytes were incubated with 200 μM BT2 and 100 μM 8-Br-cAMP in glucose-free DMEM containing gluconeogenic substrates (1 mM sodium pyruvate and 10 mM sodium lactate) for 24 h. The cell culture supernatants were collected to measure glucose content. **G** Volcano plots showed the differentially expressed genes between BT2-treated and control hepatocytes. **H** The top 25 most enriched pathways in KEGG pathway analysis. **I** Enrichment plot of Glycolysis/Gluconeogenesis pathway in GSEA analysis. **J**–**L** mRNA expressions of gluconeogenic genes in primary mouse hepatocytes treated with 200 μM BT2 and 100 μM 8-Br-cAMP. **M** PEPCK promoter activity was detected in HepG2 cells. **N** Protein expression of PEPCK in primary mouse hepatocytes treated with 200 μM BT2 and 100 μM 8-Br-cAMP. Data are expressed as means ± SEM for three independent experiments. ^*^P < 0.05, ^**^P < 0.01, ^**^*P < 0.001 vs control group.

It is well known that fasting-mediated activation of adenylyl cyclase leads to an increase in intracellular cyclic AMP (cAMP) concentration. As a second messenger, cAMP initiates the transcription of gluconeogenesis-related genes to maintain blood glucose levels during starvation [28–30]. In primary mouse hepatocytes, 8-Br-cAMP treatment increased BCKDK protein expression without changing its mRNA level (Fig. 4A, B). The elevation of BCKDK protein level became significantly pronounced 4 h after 8-Br-cAMP treatment (Fig. 4C). Moreover, the degradation of BCKDK protein was markedly prevented by 8-Br-cAMP in the presence of protein synthesis inhibitor CHX (Fig. 4D). These data indicate that cAMP stabilizes hepatic BCKDK protein. We further investigated the effect of BCKDK on gluconeogenesis in vitro and found that 8-Br-cAMP-stimulated hepatic glucose production was significantly decreased by BT2 incubation (Fig. 4E, F). In order to characterize the comprehensive gene expression pattern regulated by BCKDK, RNA-seq analysis was performed in primary mouse hepatocytes treated with or without 200 μM BT2 for 8 h in the presence of 100 μM 8-Br-cAMP. By setting a threshold for differential expression at fold-change ≥1.5-fold, there were 138 differentially expressed genes (76 upregulated genes and 62 downregulated genes, including classical gluconeogenic genes PEPCK and G6Pc) between BT2-treated and control cells (Fig. 4G). The downregulated genes were subjected to KEGG pathway analysis, showing that the gluconeogenesis pathway was one of the most enriched pathways in the set of genes (Fig. 4H). By using GSEA, differentially expressed genes were core enrichment in gluconeogenesis pathways (Fig. 4I). RT-qPCR analysis further demonstrated that cAMP-elicited mRNA expressions of three key gluconeogenic enzymes were significantly attenuated by BT2 treatment (Fig. 4J–L). Additionally, BT2 antagonized 8-Br-cAMP-stimulated PEPCK promoter activity (Fig. 4M) as well as its protein expression (Fig. 4N). These findings suggest that BCKDK is essential for cAMP-stimulated hepatic gluconeogenesis.

### Effect of BCKDK knockdown or overexpression on hepatic gluconeogenesis in vitro

**Fig. 5:**
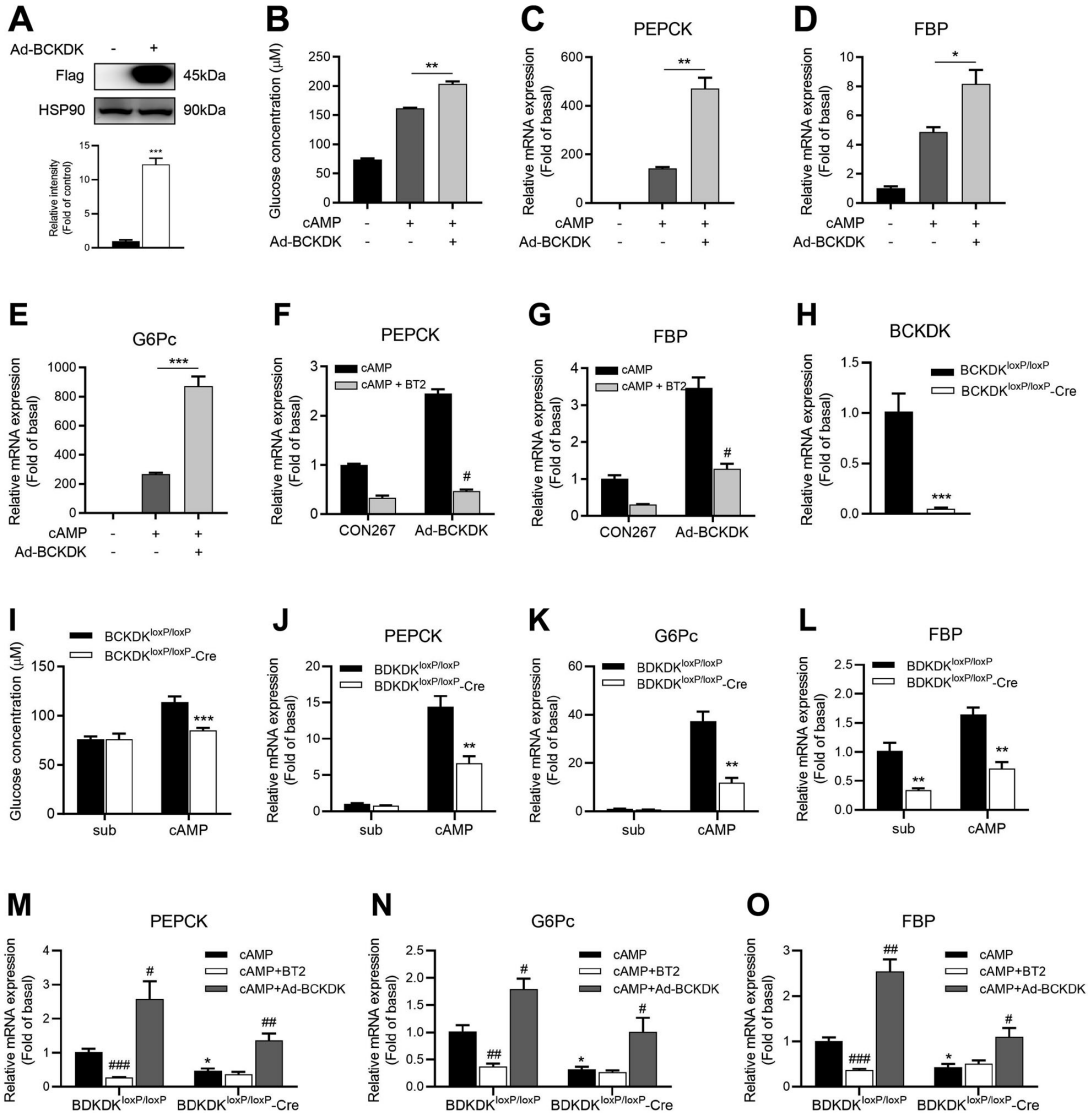
BCKDK affects the transcription of key gluconeogenic genes. **A** Protein level of Flag-BCKDK in primary mouse hepatocytes transfected with control vector (CON267) or BCKDK-overexpressing adenovirus (Ad-BCKDK). **B** After transfected with CON267 or Ad-BCKDK adenovirus, primary mouse hepatocytes were incubated with 100 μM 8-Br-cAMP in glucose-free DMEM containing gluconeogenic substrates for 24 h, and glucose content was measured. **C**–**E** mRNA levels of gluconeogenic genes in primary mouse hepatocytes transfected with Ad-BCKDK. **F**, **G** After transfected with Ad-BCKDK or CON267, primary mouse hepatocytes were incubated with 200 μM BT2 and 100 μM 8-Br-cAMP for 16 h, and PEPCK and FBP mRNA expressions were detected by RT-qPCR. **H**–**L** Primary hepatocytes isolated from BCKDK^loxP/loxP^ mice were infected with Ad-Cre and then incubated with 100 μM 8-Br-cAMP for 16 h, hepatic glucose production as well as mRNA expressions of BCKDK and gluconeogenic genes were assayed respectively. **M**–**O** mRNA levels of three key gluconeogenic enzymes in BCKDK^loxP/loxP^ hepatocytes co-transfected with Ad-Cre and Ad-BCKDK and incubated with 100 μM 8-Br-cAMP and 200 μM BT2. Data are expressed as means ± SEM for three independent experiments. ^*^P < 0.05, ^**^P < 0.01, ^***^P < 0.001 vs control group. ^#^*P* < 0.05, ^##^*P* < 0.01, ^###^*P* < 0.001 vs cAMP group.

To further explore the regulatory role of BCKDK in gluconeogenesis, we transfected BCKDK-overexpressing adenovirus (Ad-BCKDK) into isolated primary mouse hepatocytes. After enforced expression of BCKDK (Fig. 5A), hepatic glucose production was markedly enhanced in the presence of 8-Br-cAMP (Fig. 5B) with increased expressions of PEPCK, FBP, and G6Pc (Fig. 5C–E). Moreover, BT2 antagonized BCKDK overexpression-elevated mRNA levels of PEPCK and FBP (Fig. 5F, G). We deleted BCKDK gene expression specifically using a Cre-loxP-based recombination system [31]. Primary hepatocytes isolated from mice homozygous for a floxed BCKDK allele (BCKDK^loxP/loxP^) were infected with adenovirus expressing the Cre-recombinase gene (Ad-Cre), leading to a 95% reduction of BCKDK mRNA expression (Fig. 5H). In Ad-Cre-infected (BCKDK^loxP/loxP^-Cre) hepatocytes, hepatic glucose production and expressions of three key gluconeogenic enzymes were significantly decreased (Fig. 5I–L), which could be reversed by Ad-BCKDK. But BT2 failed to exhibit a synergistic inhibitory effect (Fig. 5M–O).

### BCKDK regulates the expression of gluconeogenic enzymes independent of BCKDHA

**Fig. 6:**
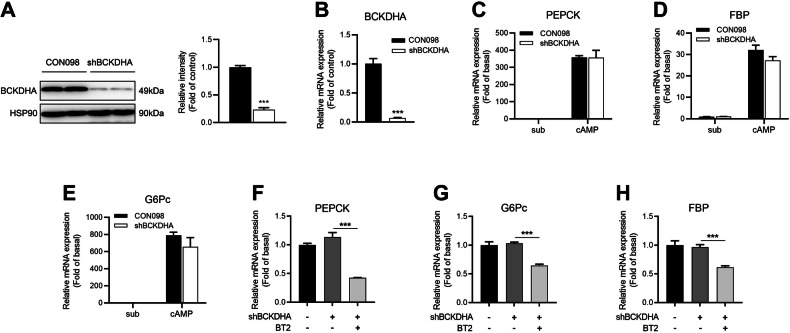
BCKDK regulates hepatic gluconeogenesis independent of BCKDHA. **A**–**B** Protein and mRNA levels of BCKDHA in primary mouse hepatocytes transfected with control vector (CON098) or shRNA targeting BCKDHA (shBCKDHA). **C**–**E** After transfected with shBCKDHA or CON098, primary mouse hepatocytes were incubated with 100 μM 8-Br-cAMP for 16 h, mRNA expressions of three gluconeogenic enzymes were detected by RT-qPCR. **F**–**H** mRNA levels of gluconeogenic genes in primary mouse hepatocytes transfected with shBCKDHA in the presence or absence of 200 μM BT2 treatment. Data are expressed as means ± SEM for three independent experiments. ^***^P < 0.001 vs control group.

Our in vivo study found that hepatic knockout of BCKDHA had no effect on gluconeogenesis. To determine whether BCKDHA was involved in BCKDK-mediated regulation of hepatic glucose production in vitro, primary mouse hepatocytes were transfected with control shRNA (CON098) or shRNA targeting BCKDHA (shBCKDHA). Following the substantial decrease of BCKDHA protein and mRNA expressions by shBCKDHA (Fig. 6A, B), no significant alterations were observed in the expressions of gluconeogenic genes, including PEPCK, FBP, and G6Pc (Fig. 6C–E). Furthermore, the inhibitory effect of BT2 on gluconeogenic gene expressions still remained after BCKDHA knockdown (Fig. 6F–H), suggesting that BCKDK regulates the expression of gluconeogenic enzymes through a pathway independent of BCDKHA.

### BT2 inhibits CREB transcriptional activity by dissociating CBP from CREB

**Fig. 7:**
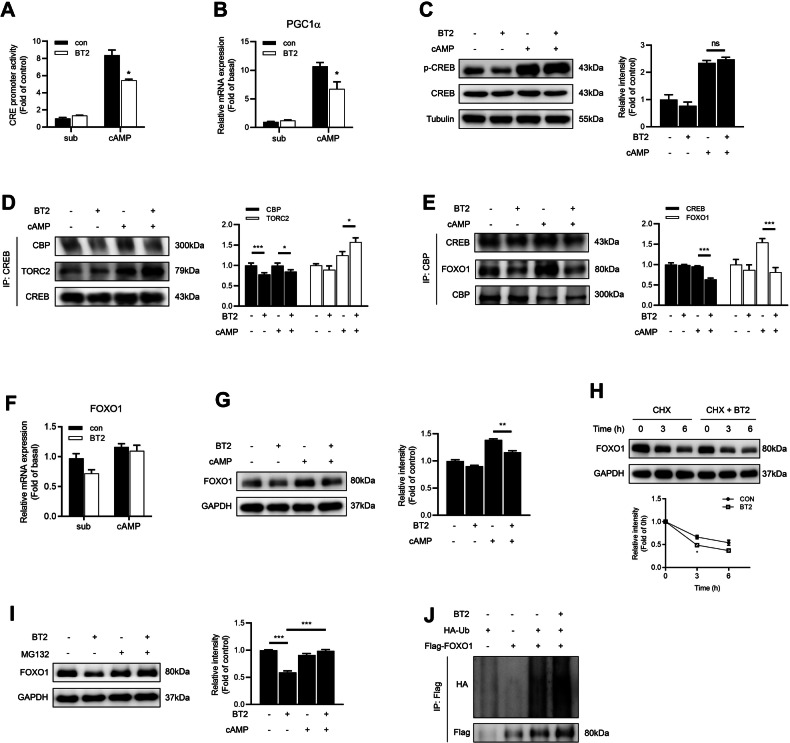
BCKDK inhibition leads to the dissociation of CBP from CREB and destabilizes FOXO1 protein via promoting its ubiquitination. **A** CRE promoter activity was detected in HepG2 cells treated with or without 100 μM 8-Br-cAMP and 200 μM BT2. **B** mRNA expression of PGC1α in primary mouse hepatocytes treated with 100 μM 8-Br-cAMP and 200 μM BT2. **C** Primary mouse hepatocytes were incubated with 100 μM 8-Br-cAMP and 200 μM BT2 for 1 h. The phosphorylation level of CREB was detected by western blot. **D** The interaction between CREB and CBP was detected in HepG2 cells by coimmunoprecipitation (CoIP). **E** Effect of BT2 on the interaction of CBP with CREB and FOXO1 in HepG2 cells. **F**–**G** mRNA and protein expressions of FOXO1 in primary mouse hepatocytes treated with 100 μM 8-Br-cAMP and 200 μM BT2. **H** FOXO1 protein level in primary mouse hepatocytes treated with 200 μM BT2 and 10 μg/ml CHX for the indicated time in the presence of 100 μM 8-Br-cAMP. Signal intensity was quantified by image J software for statistical comparison. **I** FOXO1 protein level in primary mouse hepatocytes treated with 200 μM BT2 and 10 μM MG132 in the presence of 100 μM 8-Br-cAMP. **J** HepG2 cells co-expressed with Flag-tagged FOXO1 and HA-tagged ubiquitin were treated with 200 μM BT2 for 6 h. FOXO1 ubiquitination level was detected. Data are expressed as means ± SEM for three independent experiments. ^*^P < 0.05, ^***^P < 0.001 *vs* corresponding control group.

cAMP response element binding protein (CREB) is a key transcription factor for gluconeogenic genes. In the present study, BT2 decreased the cAMP response element (CRE) luciferase activity stimulated by 8-Br-cAMP in HepG2 cells (Fig. 7A). In addition, cAMP-enhanced mRNA expression of PGC1α, a gluconeogenic coactivator targeted by CREB, was downregulated by BT2 correspondingly (Fig. 7B). It is possible that BT2 represses the expression of gluconeogenic enzymes by affecting the transcriptional activity of CREB. However, BT2 treatment did not alter cAMP/PKA-mediated phosphorylation level of CREB at Ser133 (Fig. 7C). It has been demonstrated that phosphorylated CREB recruits coactivators such as CREB-binding protein (CBP) and CREB-regulated transcription coactivator 2 (TORC2) to CRE containing genes and facilitates gluconeogenesis [32, 33]. Here cAMP-induced recruitment of CBP to CREB was dramatically impaired in the presence of BT2 (Fig. 7D, E). Collectively, these results indicate that BCKDK inhibition decreases CREB transcriptional activity by dissociating CBP from CREB.

### BCKDK inhibition destabilizes FOXO1 protein by promoting its ubiquitination

Forkhead box O1 (FOXO1) is another transcription factor tightly linked to hepatic gluconeogenesis [34]. The histone acetyltransferase (HAT) domain of CBP activates FOXO1 acetylation and regulates its protein stability [35, 36]. In this current study, the interaction between FOXO1 and CBP was also attenuated by BT2 (Fig. 7E). Though BT2 had no effect on FOXO1 mRNA expression (Fig. 7F), it antagonized cAMP-elevated FOXO1 protein expression (Fig. 7G). In addition, BT2 shortened the half-life of FOXO1 protein in the presence of CHX (Fig. 7H) while BT2-induced decrease in FOXO1 protein expression could be readily prevented by the proteasome inhibitor MG132 (Fig. 7I). To address whether BCKDK inhibition targets FOXO1 protein to proteasomal degradation, Flag-FOXO1 together with HA-Ub expression plasmids were co-transfected into HepG2 cells. After treatment with MG132, the ubiquitination level of Flag-FOXO1 was obviously increased in cells exposed to BT2 as detected by HA antibody (Fig. 7J).

## Discussion

Recent studies in rodents and humans have shown that pharmacological inhibition of BCKDK significantly improved insulin sensitivity [8, 14–16, 37], providing a novel therapeutic target for metabolic diseases. Enhanced hepatic gluconeogenesis is a crucial contributor to hyperglycemia in type 2 diabetes. The present study highlighted the important role of BCKDK in the regulation of gluconeogenesis in vivo by using liver-specific BCKDK knockout mice. However, liver-specific deletion of BCKDHA had no impact on hepatic glucose production. Pharmacological inhibition or genetic deletion of BCKDK decreased gluconeogenesis in primary mouse hepatocytes by inhibiting the expressions of key gluconeogenic enzymes, which were involved in the dissociation of CBP from CREB and protein degradation of FOXO1.

It has long been recognized that elevated plasma BCAA concentrations strongly correlate with insulin resistance and obesity [6, 7], and even predict the development of type 2 diabetes in the future [11]. The activity or expression of key enzymes involved in BCAA catabolism is a major determinant of circulating BCAA levels. BCKDHA has been identified as one of the most likely candidate genes for T2DM [38]. Increased plasma levels of BCAAs have been demonstrated to be the result of decreased BCAT expression or lower BCKDH complex activity due to increased expression of BCKDK in the liver and adipose tissue of obese or diabetic rodents [14, 15, 21, 23, 39]. In this current study, BCKDK protein expression and BCKDHA phosphorylation levels were markedly increased in the liver of *db*/*db* mice. Therefore, BCAA catabolic enzyme-targeted intervention becomes a crucial strategy in the treatment of T2DM. BCKDHA activation by the BCKDK inhibitors BT2 and sodium phenylbutyrate lowered plasma BCAA levels and improved insulin sensitivity in diabetic rodents or patients [8, 14, 37]. However, the dominant tissue linking BCAA catabolism to glucose homeostasis remains elusive. BCKDHA-specific deletion in brown adipose tissue impaired glucose tolerance [40], while its ablation in WAT displayed an opposite result under high-fat diet conditions [41]. Skeletal muscle-specific BCKDHA or BCKDK knockout mice maintained normal glucose tolerance [17]. In the present study, liver-specific deletion of BCKDHA or BCKDK had no impact on glucose tolerance and insulin sensitivity, consistent with the results previously reported [16, 17]. More recently, metabolic off-target effects of BT2 have been revealed [42]. Therefore, it is possible that the beneficial effects of BT2 on whole-body metabolism rely on inter-organ communication through systemic inhibition of BCKDK rather than that of a particular tissue.

The end products of BCAA catabolism (succinyl-CoA and acetyl-CoA) enter the TCA cycle and are incorporated into alternative pathways for gluconeogenesis [15]. Recently, Zhao et al. reported that enhancing BCAA metabolism by BT2 inhibited renal gluconeogenic gene expressions, without changing hepatic gluconeogenic gene expressions [43]. Inconsistent with this result, our study showed that specific knockout of BCKDK in the liver significantly decreased hepatic gluconeogenesis and key gene expressions. This discrepancy is mainly attributed to different methods of intervention in BCKDK. It is likely that the extrahepatic effects of systemic BCKDK inhibition by BT2 interfere with its direct impact on hepatic gluconeogenic gene expression. Another study pointed towards a decrease in hepatic glucose production of mice with impaired BCAA catabolism due to PP2Cm knockout, in which intracellular metabolite flux, rather than the key gluconeogenic enzymes, affected gluconeogenesis [44]. In our study, no differences were detected in pyruvate tolerance and gluconeogenic gene expressions between PP2Cm KO mice and control mice (Fig. S3). Based on the findings that liver-specific knockout of BCKDHA had no effect on gluconeogenesis in mice, we suggest that BCKDK governs hepatic glucose production through transcriptional regulation of gluconeogenic genes independent of BCAA catabolism.

To investigate whether hepatic deficiency of BCKDK exerts a beneficial effect on obese animal models, BCKDK^Alb^ KO mice were fed with HFD. Unexpectedly, BCKDK^Alb^ KO mice obtained more weight and exhibited more serious insulin resistance and pyruvate intolerance under HFD conditions. This result is inconsistent with previous studies, in which mice with liver-specific knockdown of BCKDK mediated by AAV remained parallel glucose tolerance and insulin sensitivity compared to control littermates fed HFD for 2 months [16, 17]. Different experimental conditions or approaches may account for the discrepancy. Importantly, HFD challenge in this current study lasted for up to 24 weeks, a duration rarely explored in previous studies. It is likely that prolonged exposure to HFD-indued fat mass gain exacerbates insulin resistance, and thereby diminishes the inhibitory effect of hepatic BCKDK knockout on gluconeogenesis. Accumulating evidence showed that dietary restriction of BCAAs improved glucose tolerance and insulin resistance in rodent models [45, 46]. Whereas, BCAA supplementation displayed various metabolic effects in different studies, with beneficial, adverse, or unchanged results [47–50]. It was reported that BCAA supplementation enhanced energy expenditure, decreased body weight, and fat mass, and mitigated hepatic steatosis in HFD-induced obese rodents [51–53]. Another study demonstrated that BCAA supplementation increased hepatic glucose production in HFD-fed mice [54]. In addition, each BCAA may exert unique influences on energy homeostasis. One recent study has shown that the adverse metabolic impacts of BCAAs are mediated by isoleucine and valine [47]. Leucine supplementation decreased G6Pc and PEPCK expressions in the livers of rats fed with HFD [27], and improved glucose tolerance and insulin sensitivity in HFD-fed mice [53]. Here hepatic BCKDK deficiency-mediated suppression of hepatic glucose production remained in HFD-fed mice due to BCAA supplementation biased towards leucine (leucine: isoleucine: valine = 2: 1: 1), which may be attributed to increased insulin sensitivity in the liver. Compared with HFD alone, HFD + BCAA inevitably contributes to more metabolic stress. Liver-specific BCKDK deletion granted mice a relatively greater capacity to deal with the metabolic stress caused by additional BCAAs. Thus, the differences of body weight, fat mass, and RER disappeared between HFD-fed BCKDK^Alb^ KO and control mice when BCAA diet was added. Taken together, BCAAs exert various impacts on whole energy homeostasis depending on the dietary composition and metabolic status of an organism. BCAA supplementation antagonizes the adverse metabolic effects of HFD when its catabolism is promoted in the liver.

Both hormonal and nutritional signals influence BCKDK activity. BCKDK is activated by the thyroid hormones and inactivated by the glucocorticoids [55, 56]. Animals with a low-protein diet or fructose feeding display increased BCKDK mRNA levels [14, 57], and prolonged starvation increases BCKDK activity [58]. Our study showed that fasting increased BCKDK protein expression and BCKDHA phosphorylation levels, without changing BCKDK mRNA level. In primary mouse hepatocytes, 8-Br-cAMP treatment showed a similar effect via preventing BCKDK protein degradation. Pharmacological inhibition by BT2 and genetic knockdown of BCKDK inhibited cAMP-stimulated gluconeogenesis in primary mouse hepatocytes, while BCKDHA silencing did not alter the expressions of hepatic gluconeogenic genes. Moreover, the inhibitory effect of BT2 on gluconeogenic gene expression remained in the case of BCKDHA knockdown, further confirming the hypothesis that BCKDK regulates hepatic gluconeogenesis independently of BCKDHA.

It is likely that some beneficial effects of BT2 are mediated via off-BCKDHA effects, as previous studies have identified additional targets of BCKDK. Newgard et al. [14] demonstrated that BCKDK elicited ATP-citrate lyase phosphorylation and promoted de novo lipogenesis, which was recognized as a new regulatory node that integrates BCAA and lipid metabolism. CREB is a pivotal regulator of the gluconeogenic program in response to both hormonal and intracellular signals. Phosphorylation of CREB at Ser133 recruits CBP and TORC2 to CRE-containing genes and then promotes the transcription of gluconeogenic genes [33]. In our study, BCKDK inhibition suppressed CREB transcriptional activity by attenuating the interaction between CBP and CREB in spite of the unchanged phosphorylation level of CREB at Ser133. FOXO1 is most tightly linked to hepatic gluconeogenesis among FOXO family members [34]. Insulin and glucagon can coordinately regulate hepatic gluconeogenesis by targeting different phosphorylation sites of FOXO1 [59–61]. In the prsent study, FOXO1 protein was found to be destabilized by BCKDK inhibition through promoting its ubiquitination. These results suggest that CREB and FOXO1, instead of BCKDHA, mediate the suppression of gluconeogenesis in hepatocytes in response to BCKDK inhibition.

In summary, BCKDK knockdown or inhibition decreases hepatic glucose production and key gluconeogenic gene expressions in vivo and in vitro, while BCKDHA knockdown is without effects. BCKDK inhibition promotes the dissociation of CBP from CREB as well as FOXO1 protein degradation. These findings indicate that BCKDK regulates hepatic gluconeogenesis through CREB and FOXO1 signaling pathways independent of BCKDHA-mediated BCAA catabolism. Therefore, liver-specific inhibition of BCKDK-mediated gluconeogenesis will provide a new strategy for the treatment of type 2 diabetes.

## Supplementary information

Supplementary figure legend and data

Original full-length western blots

## Data Availability

The authors declare that all data supporting the findings of this study are available within the article and the Supplementary Information. All other data are available from the corresponding authors upon request.
