# Supplementary material for: BCKDH kinase promotes hepatic gluconeogenesis independent of BCKDHA
Source: Cell Death Dis. 2024 Oct 10;15(10):736. doi: 10.1038/s41419-024-07071-0 (PMC11467410; doi:10.1038/s41419-024-07071-0)

**Table 1**. The primer sequences used for qRT-PCR

| Gene name | Primer sequence (Forward: 5’-3’) | Primer sequence (Reverse: 5’-3’) |
| --- | --- | --- |
| 18S | AGGCGCCTGTACTGCTTCTTTTCTT | TTTGGCACTGTTGGGGAAGTCTACC |
| BCKDK | AAGGATGTGGTGACCCTGTTAGCTG | GGATCCCAAGTCTTGACGTCAGTGT |
| BCKDHA | TTCATCCAGCCCAATGTCATCTCCG | GTAGAACTTCAGCACCTCCTCCTGG |
| G6Pc | ACTGTGGGCATCAATCTCCTC | CGGGACAGACAGACGTTCAGC |
| PEPCK | GTGCTGGAGTGGATGTTCGG | CTGGCTGATTCTCTGTTTCAGG |
| FBP | GTATCGCTGGCTCAACCAAT | TCAAGGGGATCGAAACAGAC |
| PGC1α | ATACCGCAAAGAGCACGAGAAG | CTCAAGAGCAGC-GAAAGCGTCACAG |
| FOXO1 | AAGAGCGTGCCCTACTTCAA | CTCCCTCTGGATTGAGCATC |
| PP2Cm | AAACTGTCCTGACCTTGGCCTTTCT | GGCTACCACCAGTTCAACACCATCT |

**Fig. S1. Metabolic effect of hepatic BCKDK deficiency on mice fed HFD. A** Body weight of BCKDK^Alb^ KO and WT mice under HFD condition (*n*=4). **B** Total fat mass and lean mass (*n*=4). **C** Liver weight (*n*=4). **D** Blood glucose levels during insulin tolerance tests (ITT) after 24 weeks of HFD feeding (*n*=4). **E** Blood glucose levels during pyruvate tolerance tests (PTT) after 12 weeks of HFD feeding (*n*=4). **F** Blood glucose levels during pyruvate tolerance tests (PTT) after 24 weeks of HFD feeding (*n*=4). **G-I** Oxygen consumption (VO_2_), carbon dioxide (VCO_2_) production, and respiratory exchange ratios (RER) during 24 h (*n*=4). **J** General locomotor activity during light and dark periods (*n*=4). Data are expressed as means ± SEM. **P*< 0.05 *vs* control group.

**Fig. S2. Metabolic effect of liver-specific BCKDHA knockout on mice fed HFD. A** Body weight of BCKDHA^Alb^ KO and WT mice under HFD condition (*n*=5-9). **B** Total lean mass and fat mass (*n*=4-7). **C** Blood glucose levels during intraperitoneal glucose tolerance tests (GTT) after 16 h fasting (*n*=5-8). **D** Blood glucose levels during intraperitoneal pyruvate tolerance tests (PTT) after 16 h fasting (*n*=5-8). **E-G** VO_2_, VCO_2_, and RER during 24 h (*n*=5-9). **H** General locomotor activity during light and dark periods (*n*=5-9). Data are expressed as means ± SEM. ^*^*P*< 0.05 *vs* corresponding control group.

**Fig. S3. PP2Cm knockout fails to affect hepatic glucose production under NCD condition. A** Blood glucose levels during intraperitoneal pyruvate tolerance tests (PTT) after 16 h fasting (*n*=4-5). **B** PP2cm, PEPCK, G6Pc and FBP mRNA expression in the isolated liver (*n*=4). Data are expressed as means ± SEM. ^***^*P*< 0.001 *vs* corresponding control group.

**Figure S1**
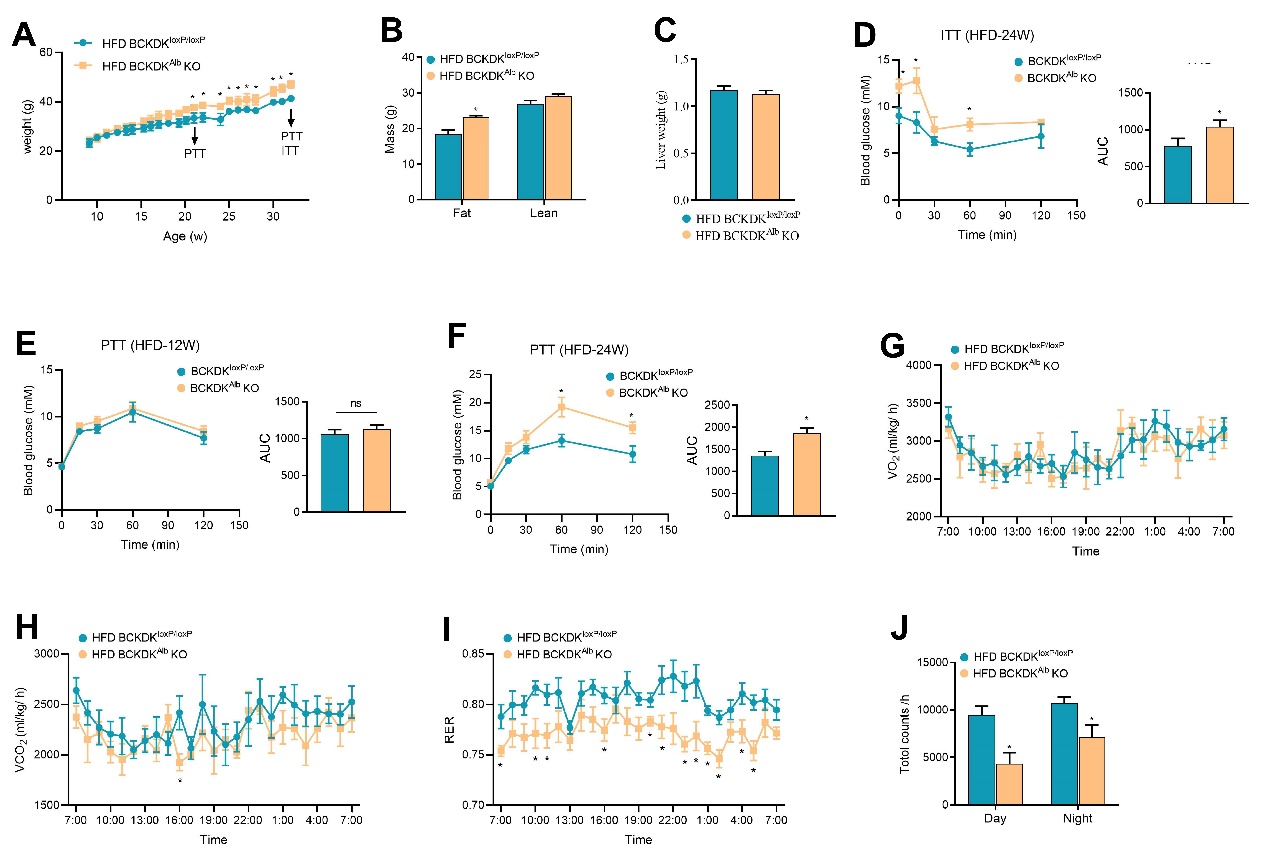


**Figure S2**

**
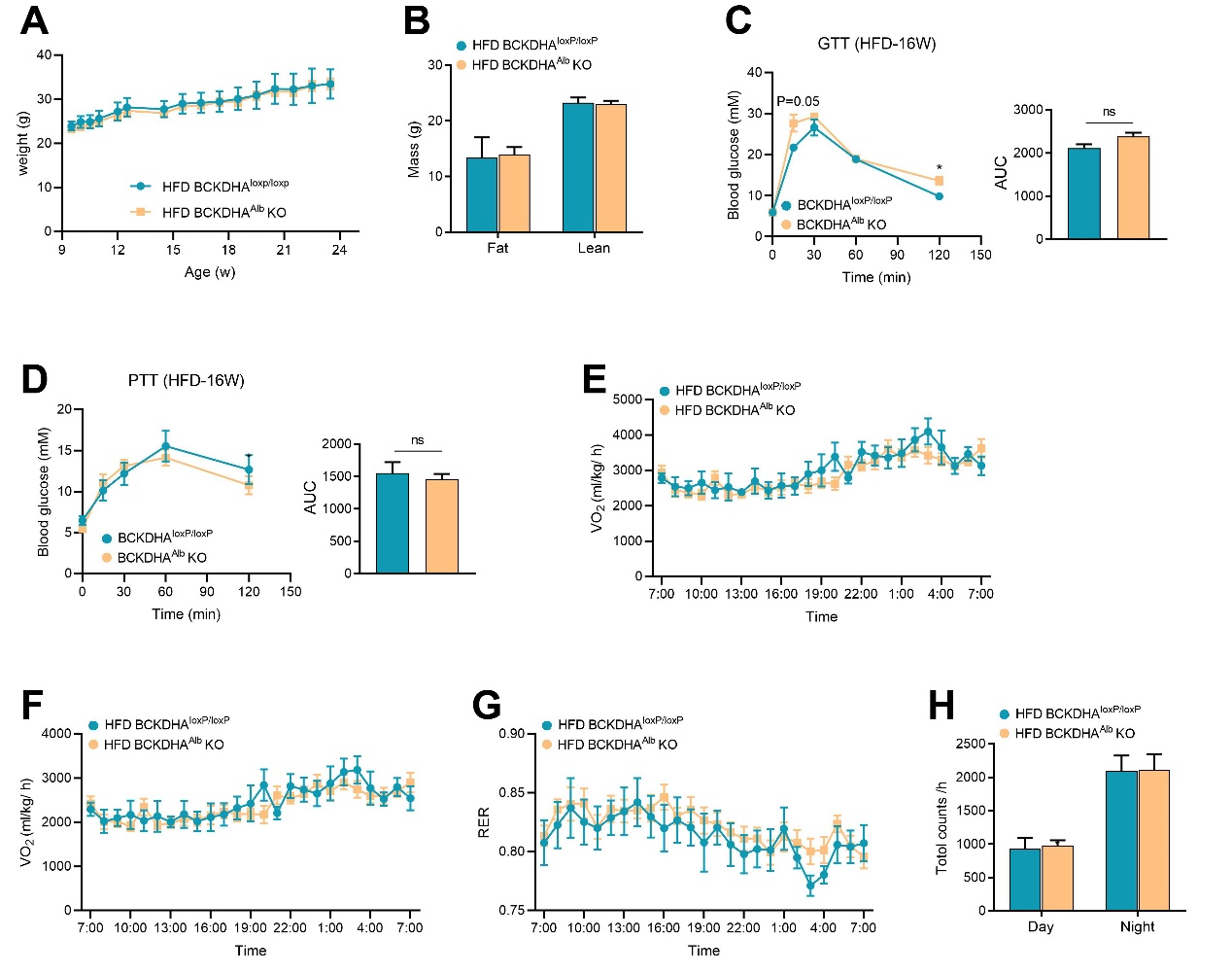
**

**Figure S3**


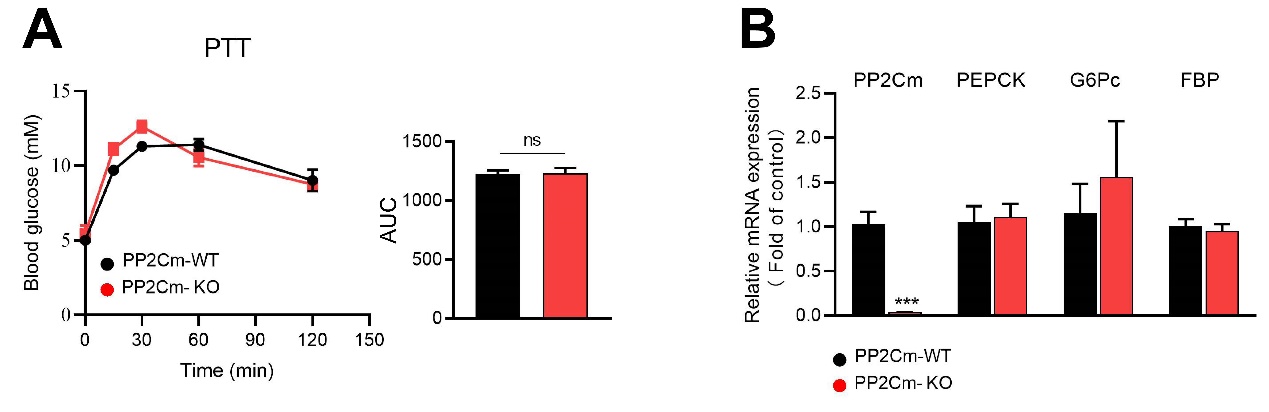

Supplement: Supplementary file 1 — Supplementary figure legend and data [file 41419_2024_7071_MOESM1_ESM.docx]
