# Supplementary material for: BCKDH kinase promotes hepatic gluconeogenesis independent of BCKDHA
Source: Cell Death Dis. 2024 Oct 10;15(10):736. doi: 10.1038/s41419-024-07071-0 (PMC11467410; doi:10.1038/s41419-024-07071-0)

Fig. 1b

BCKDK

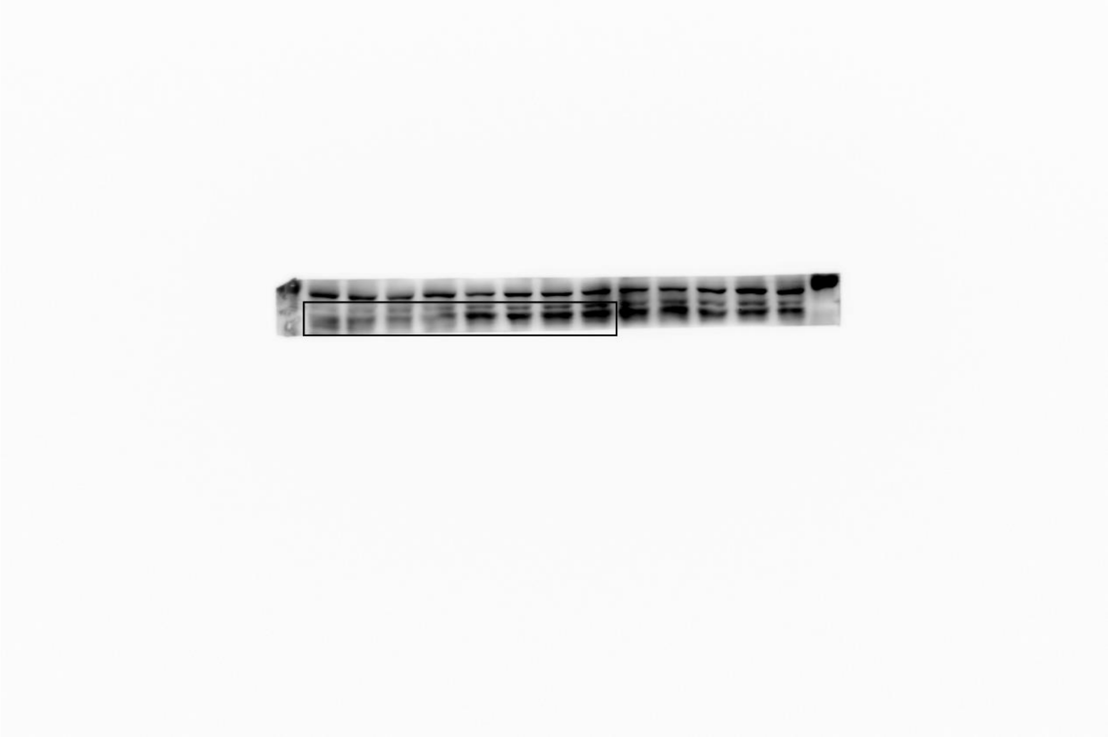

p-BCKDHA

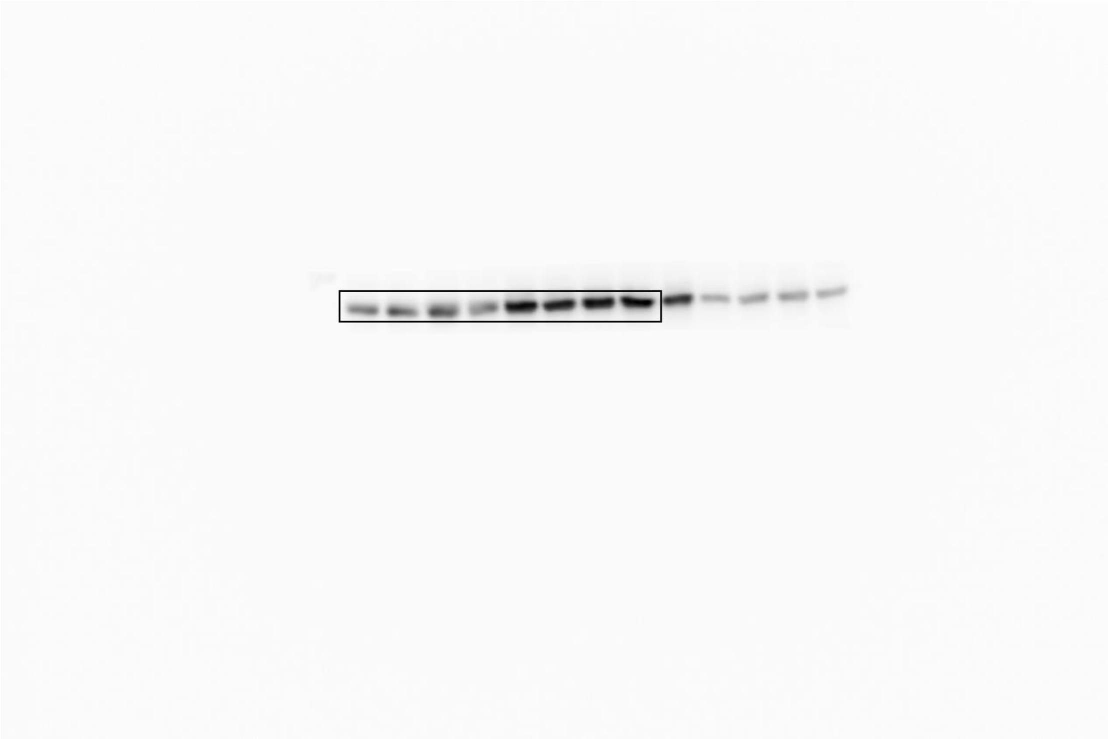

BCKDHA

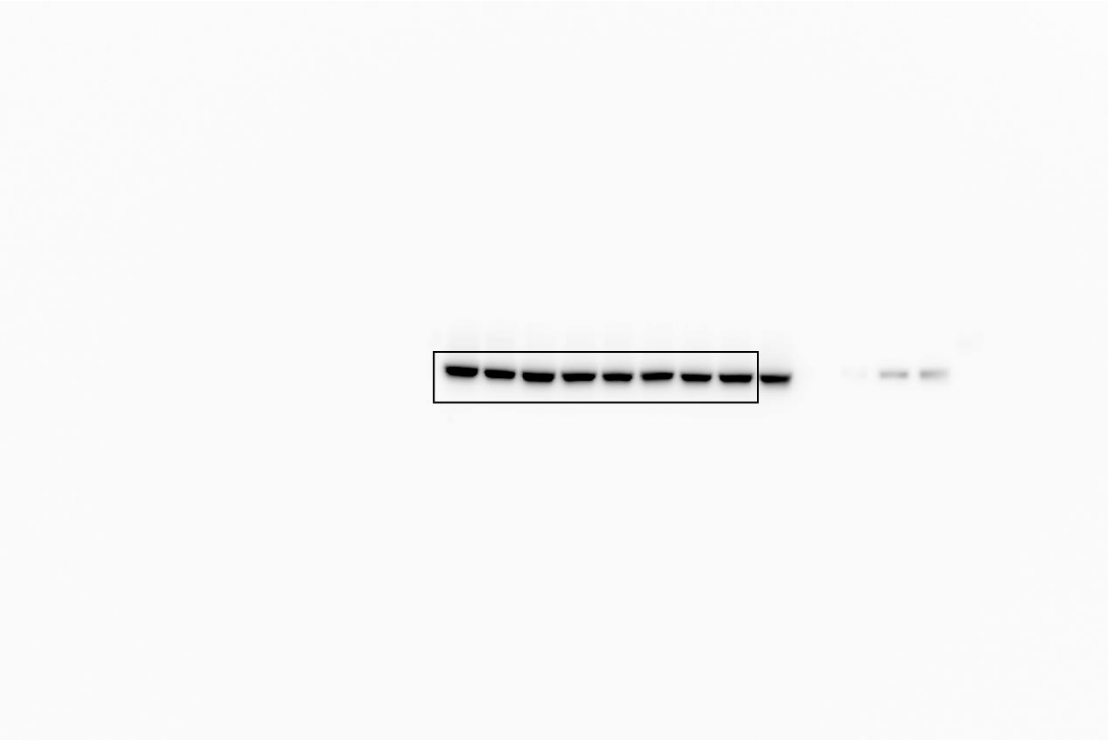

HSP90

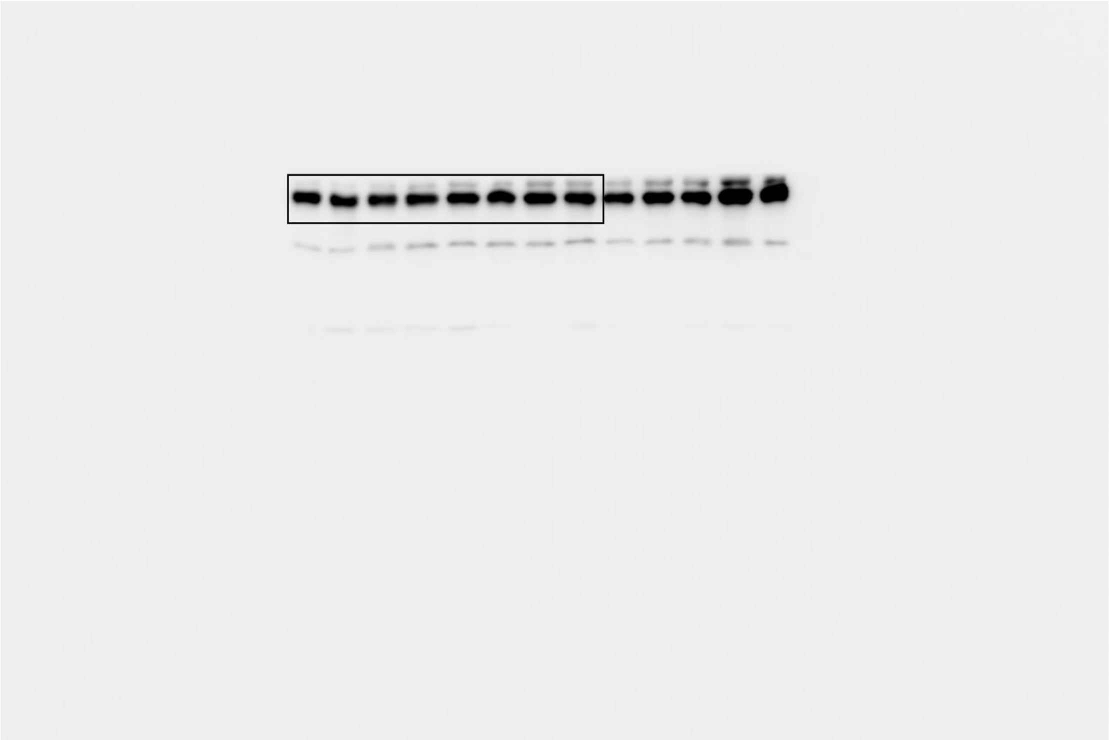

Fig. 1c

BCKDK

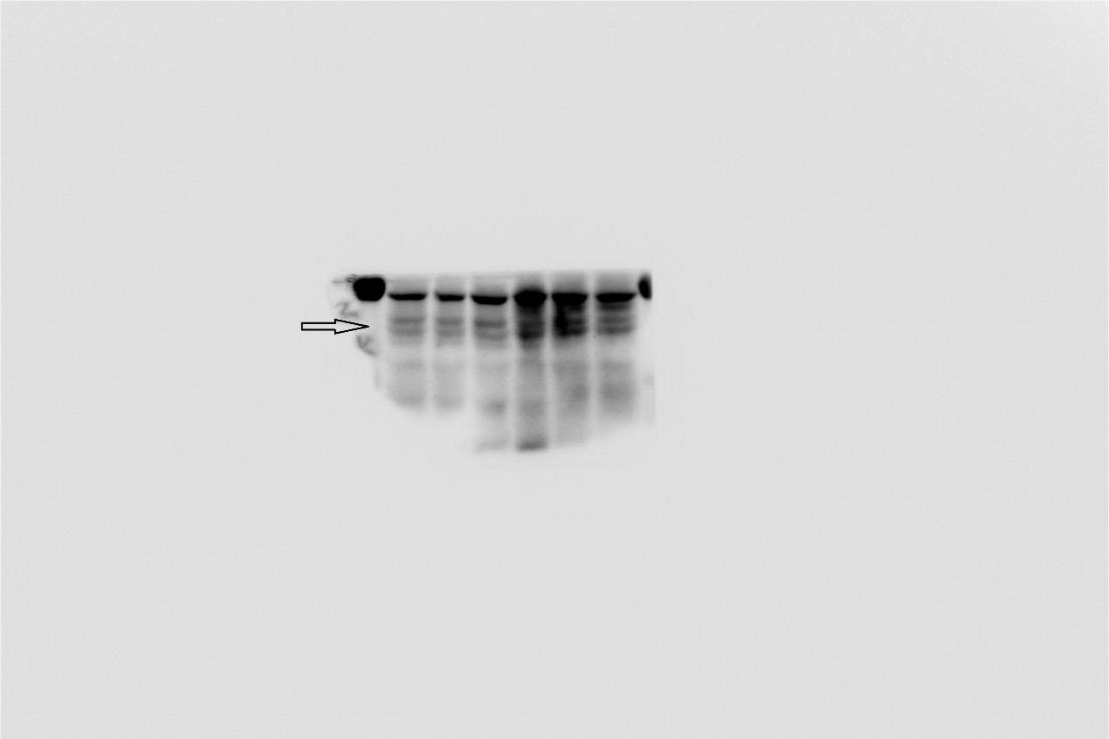

p-BCKDHA

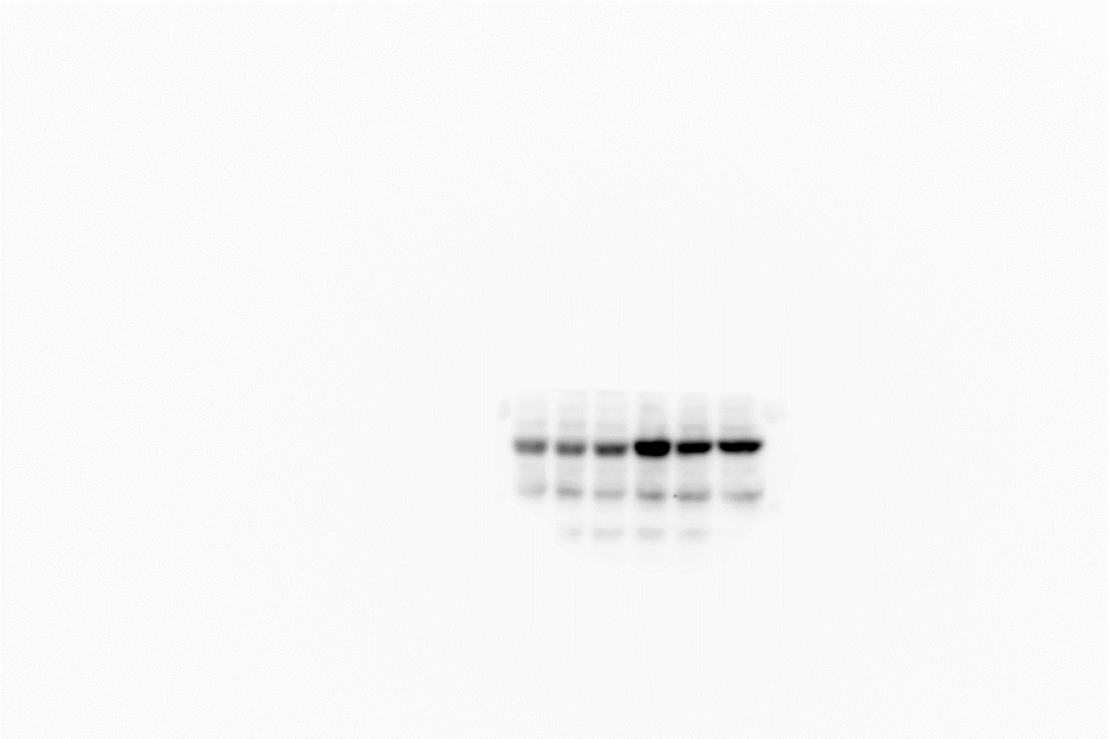

BCKDHA

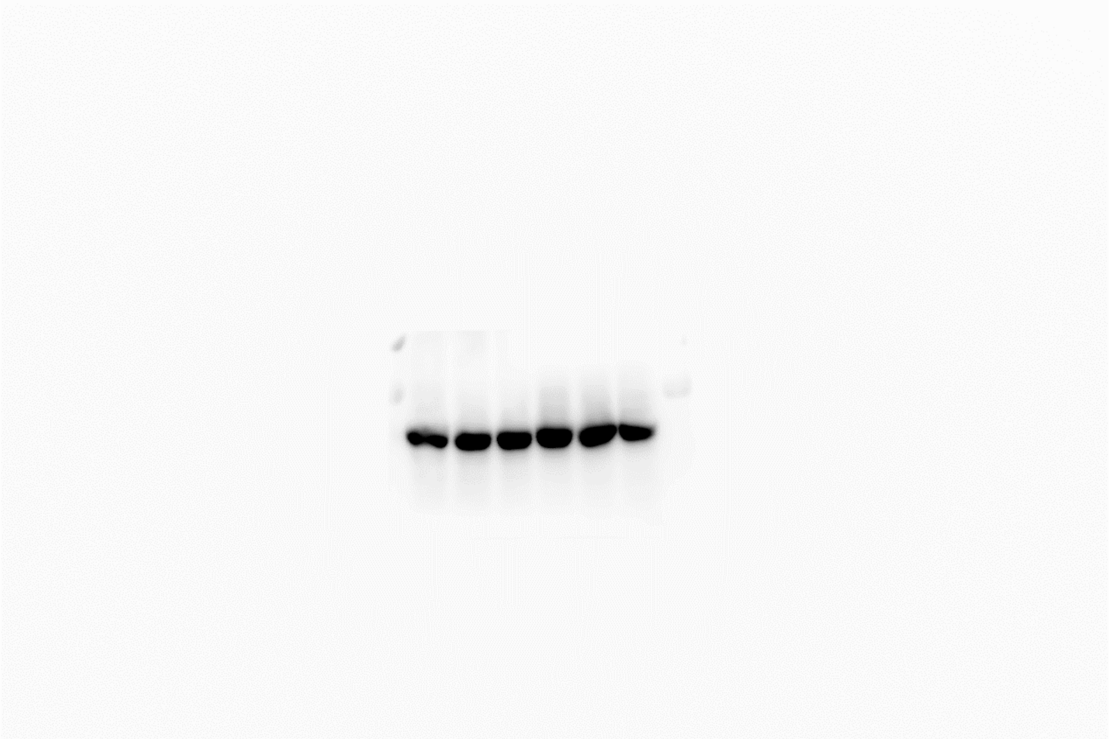

HSP90

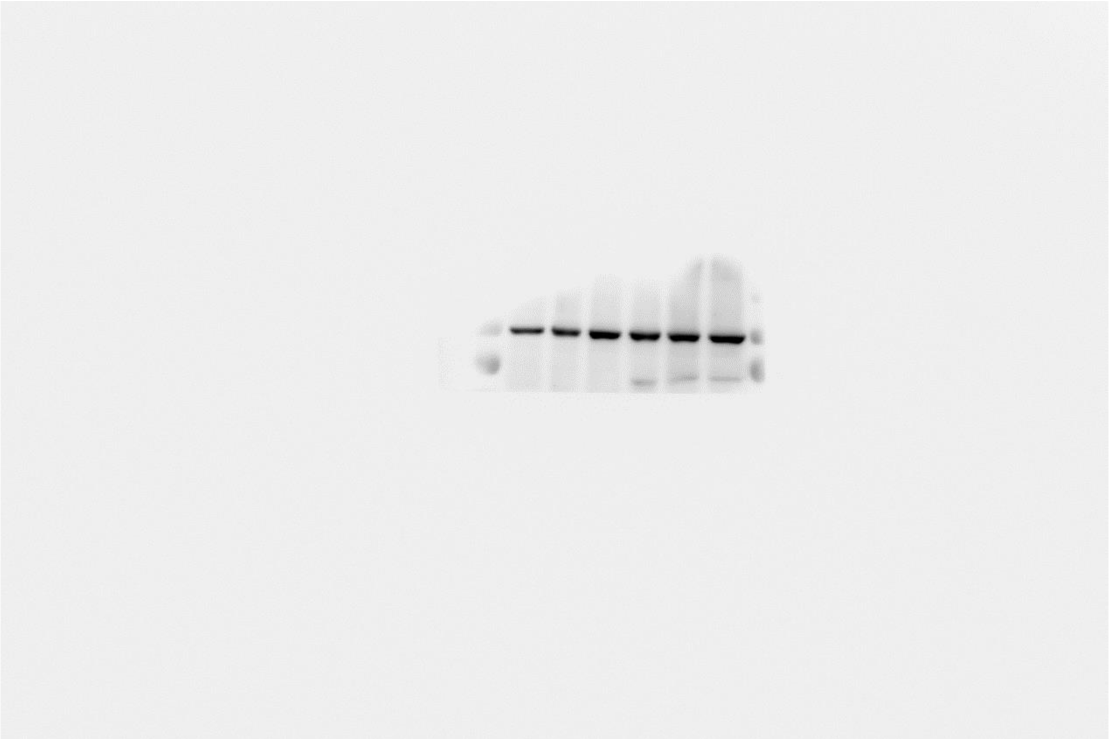

Fig. 1g

BCKDK

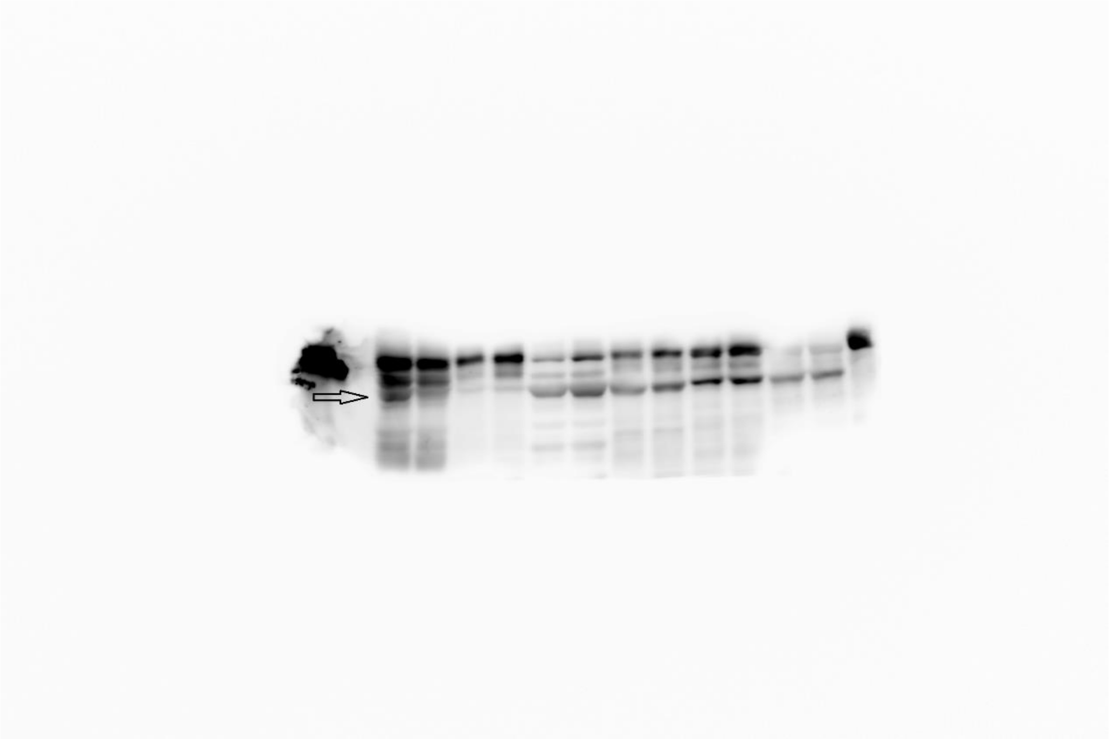

HSP90

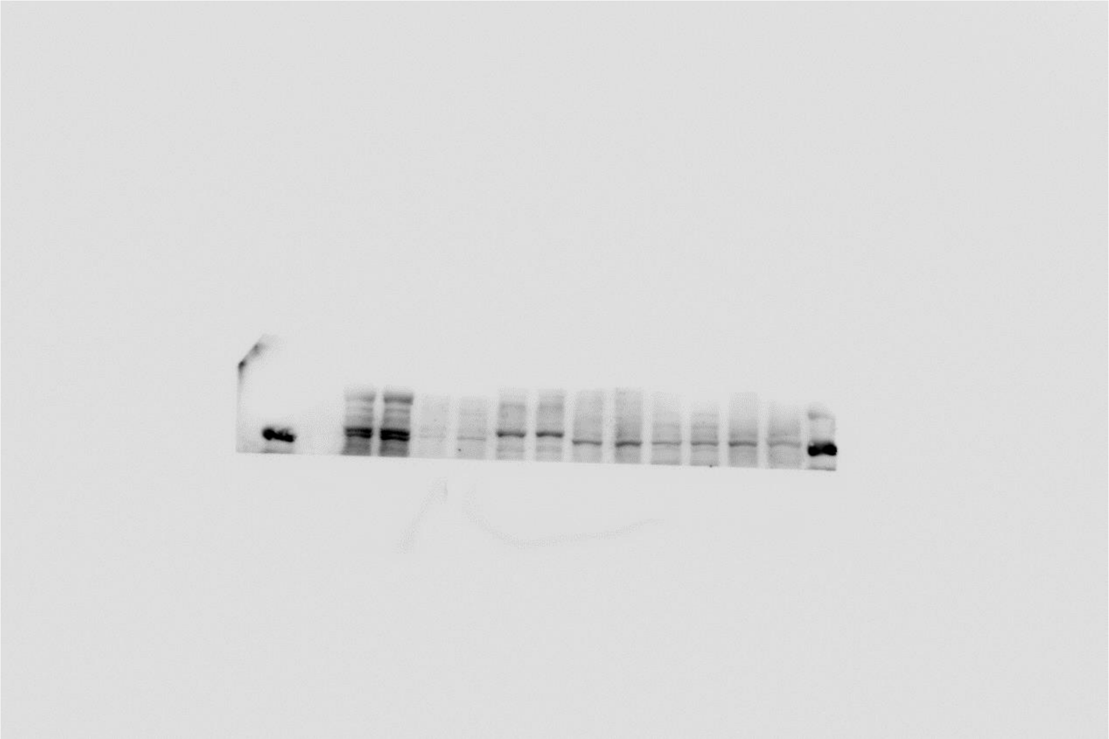

Fig. 1n

G6Pc

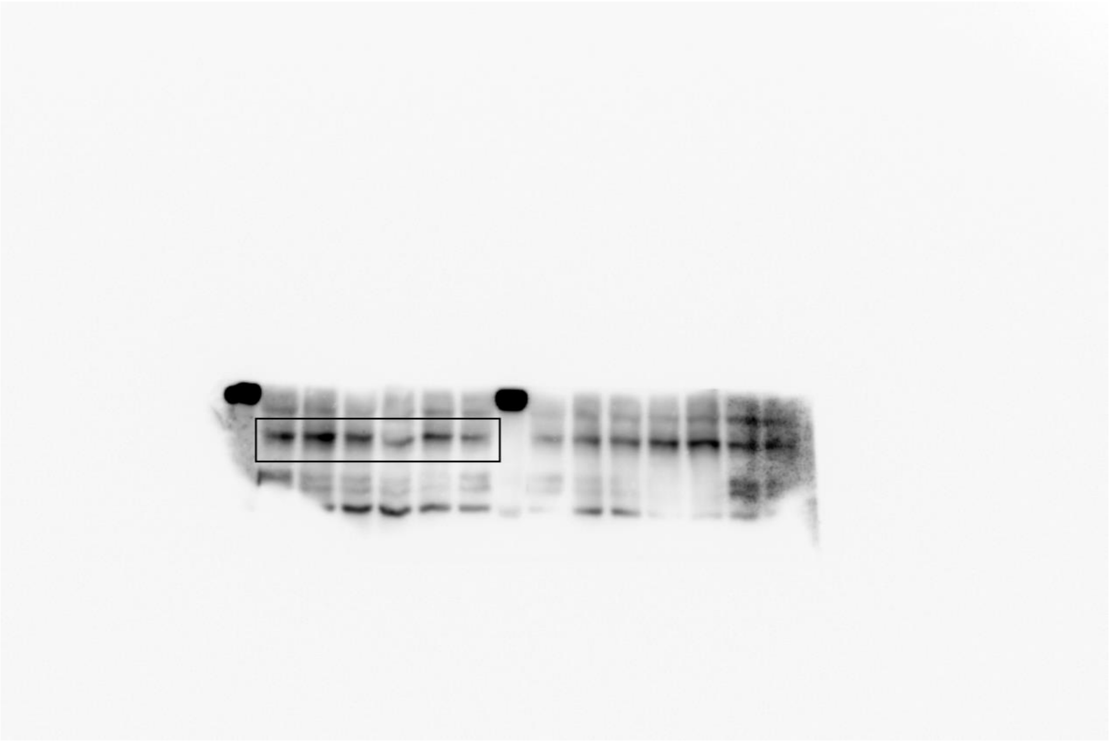

HSP90

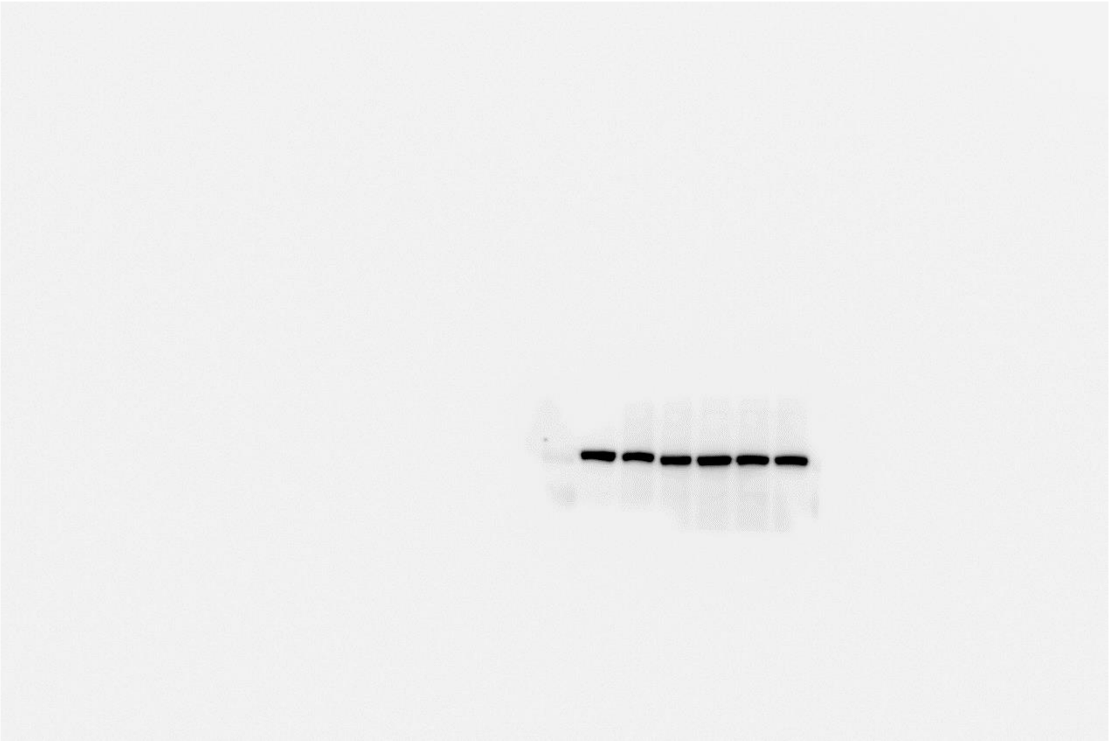

Fig. 1o

PEPCK

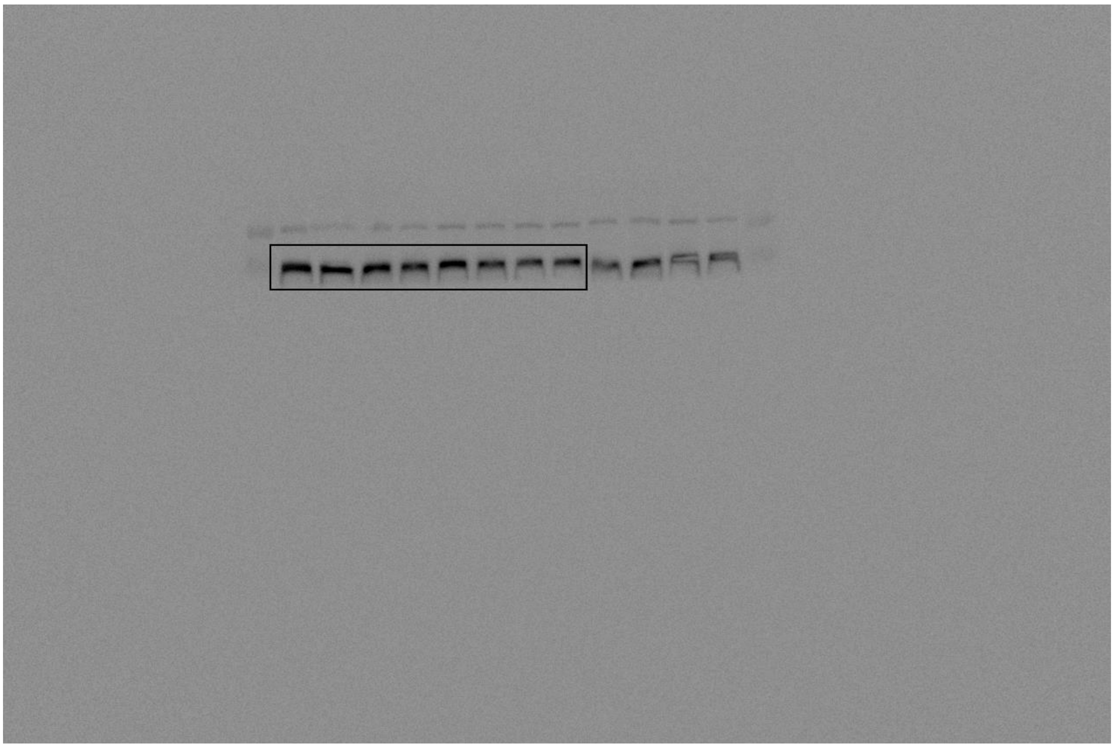

HSP90

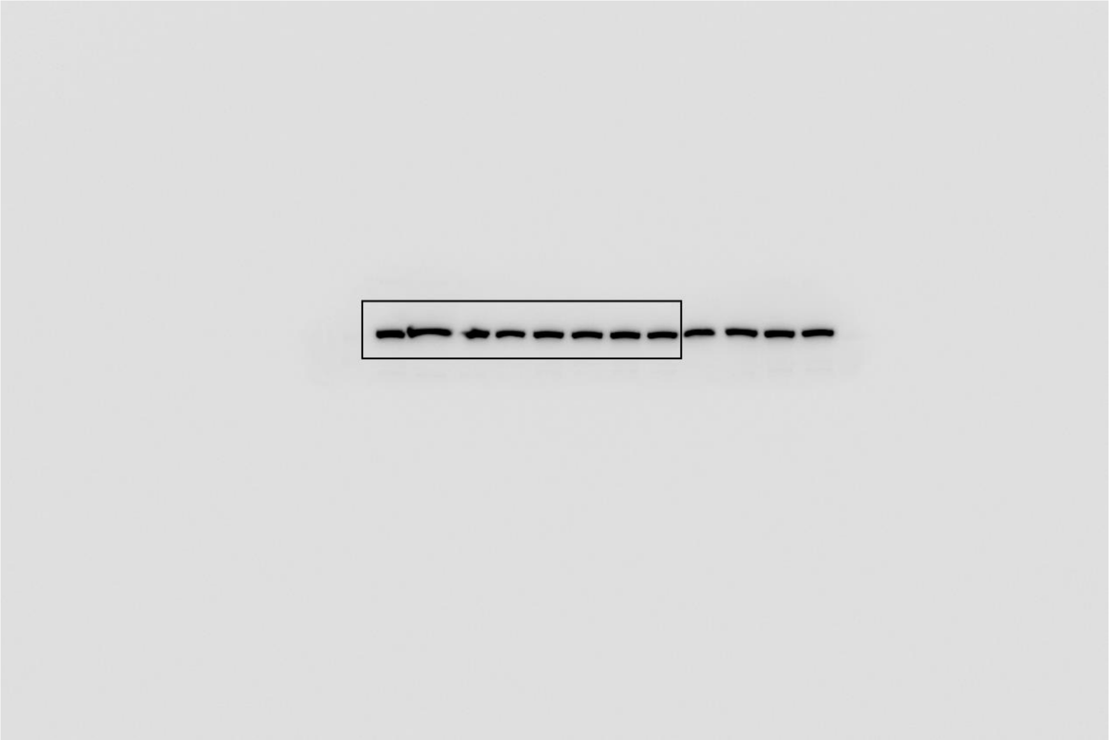

Fig. 3a

BCKDK-liver

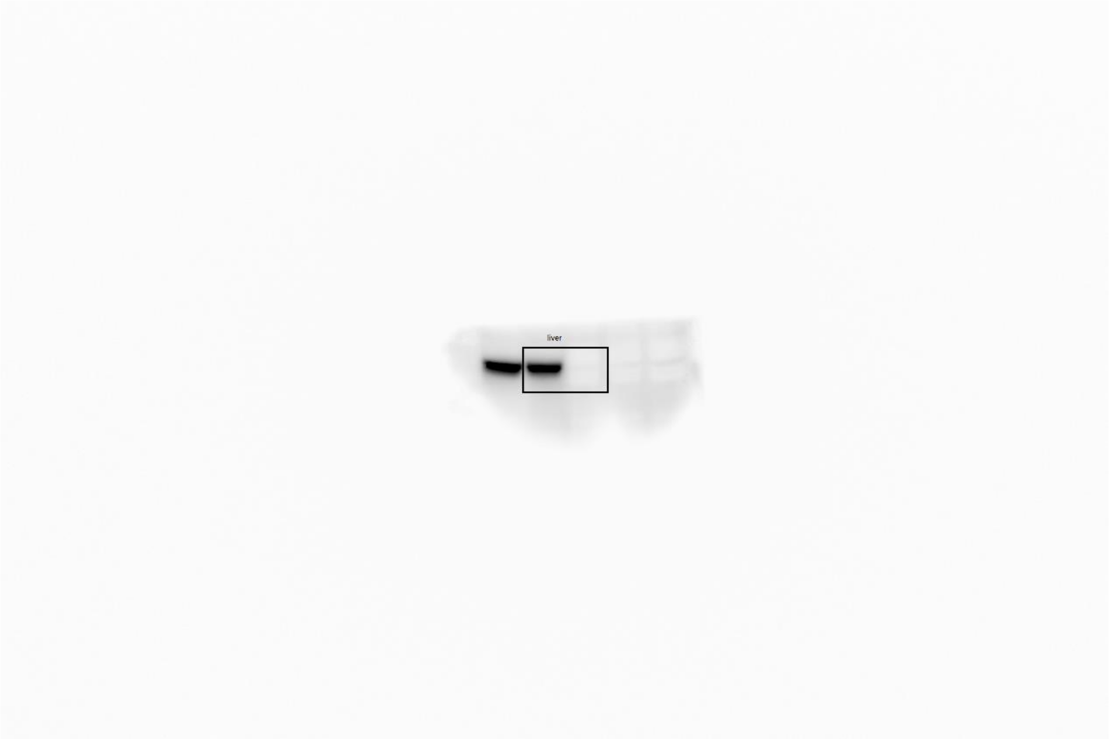

HSP90-liver

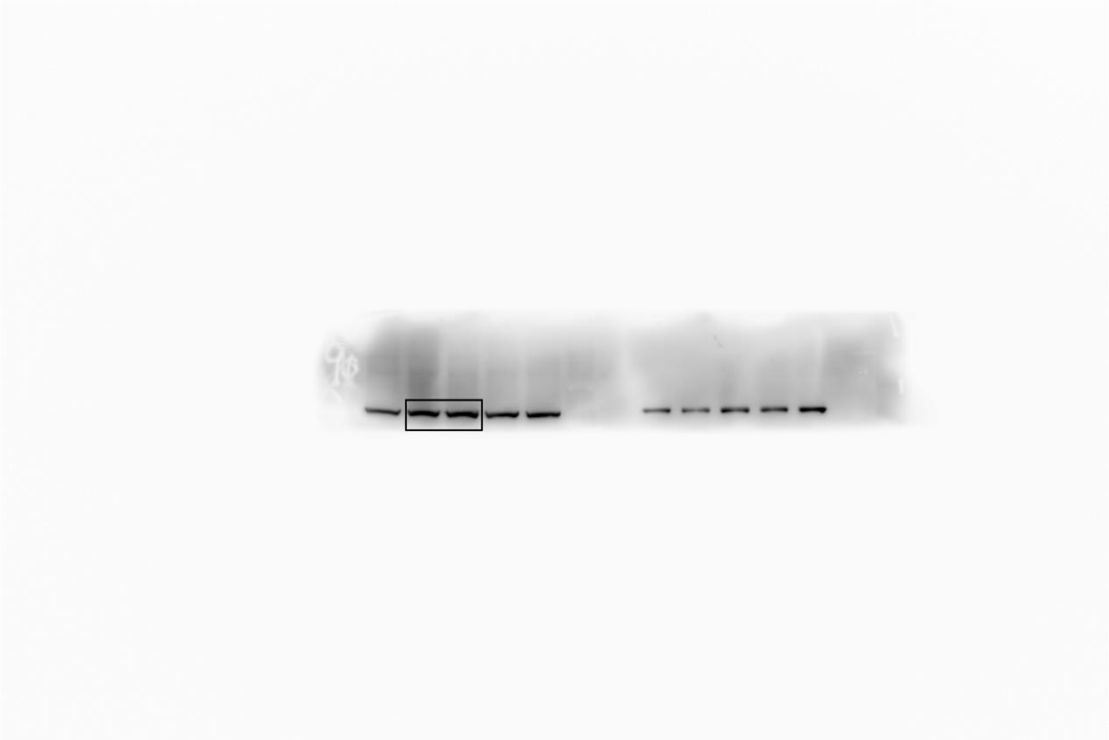

Fig. 4a

BCKDK

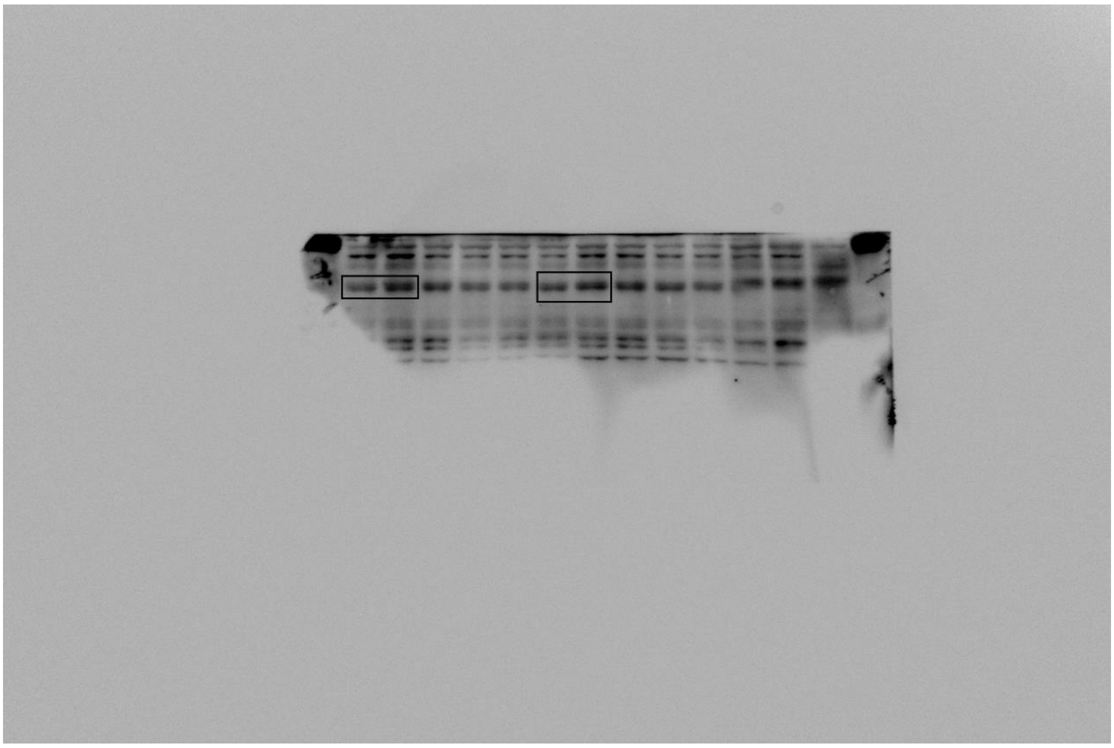

P-BCKDHA

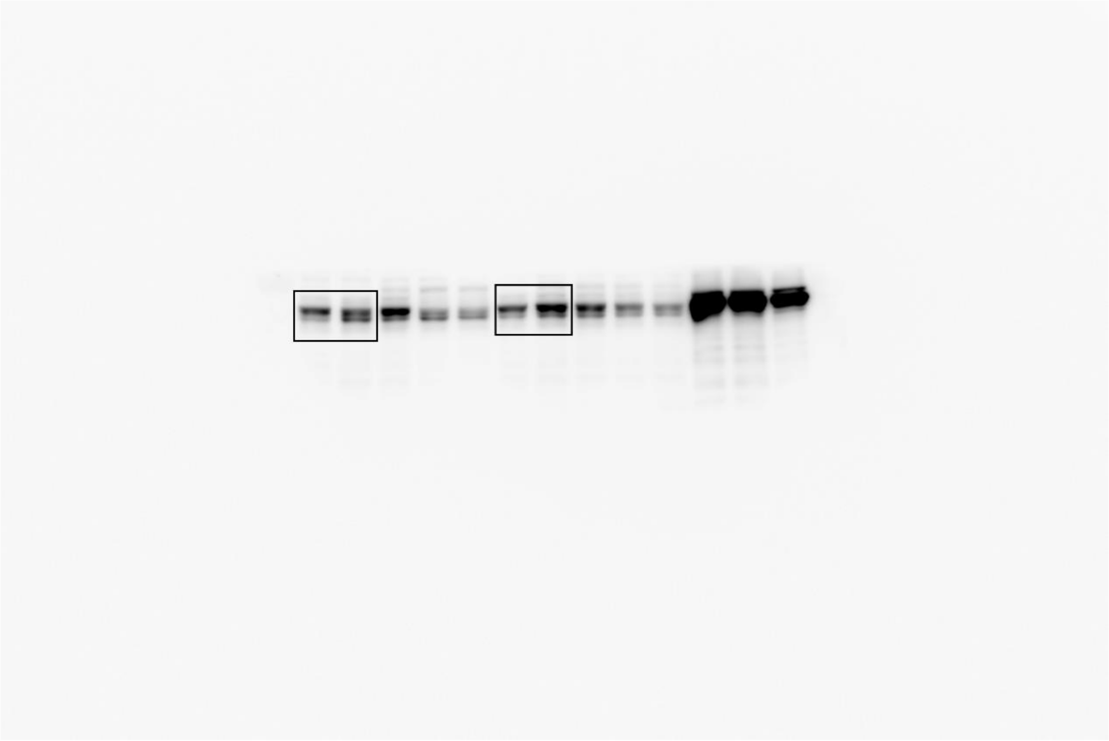

HSP90

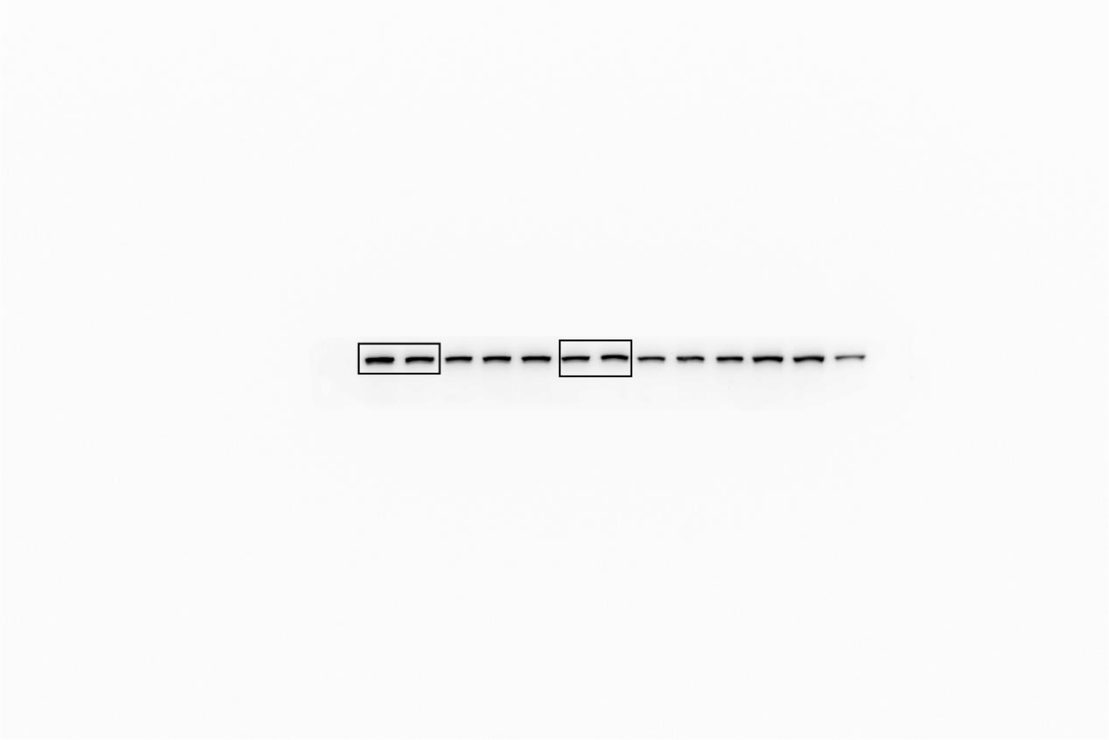

Fig. 4c

BCKDK

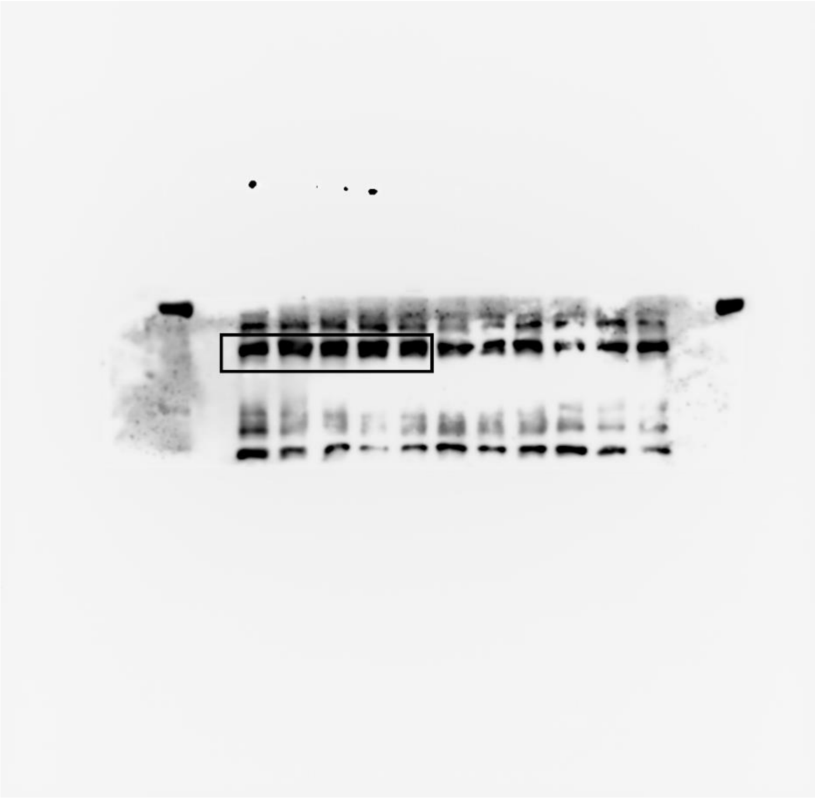

HSP90

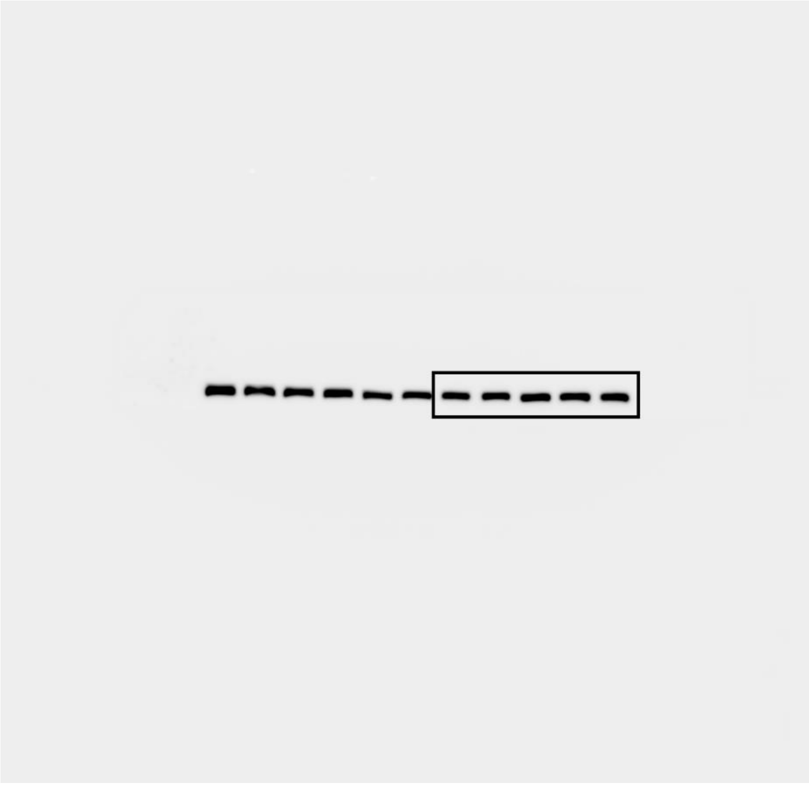

Fig. 4d

BCKDK

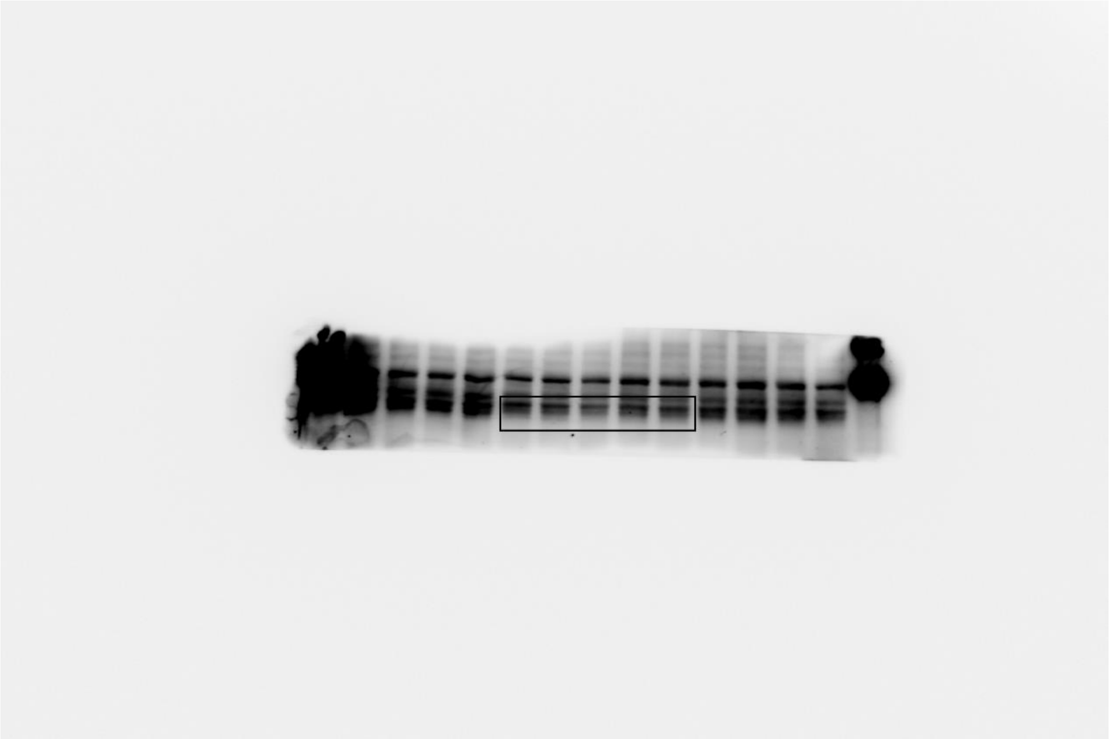

HSP90

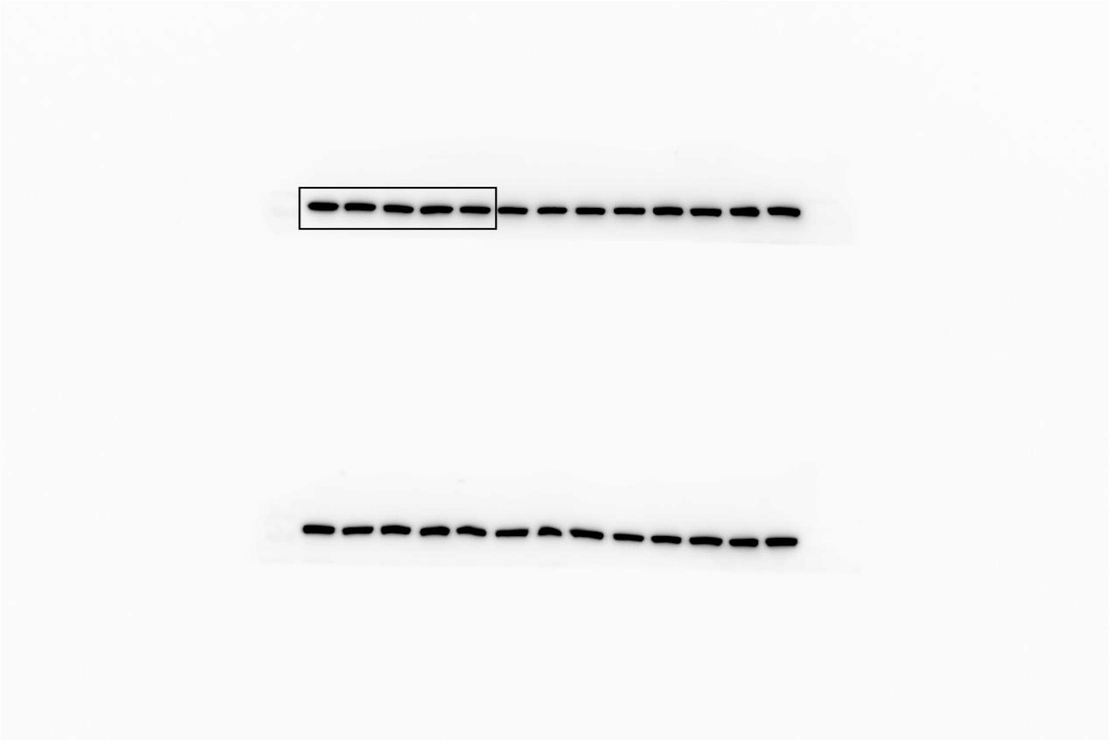

Fig. 4e

P-BCKDHA

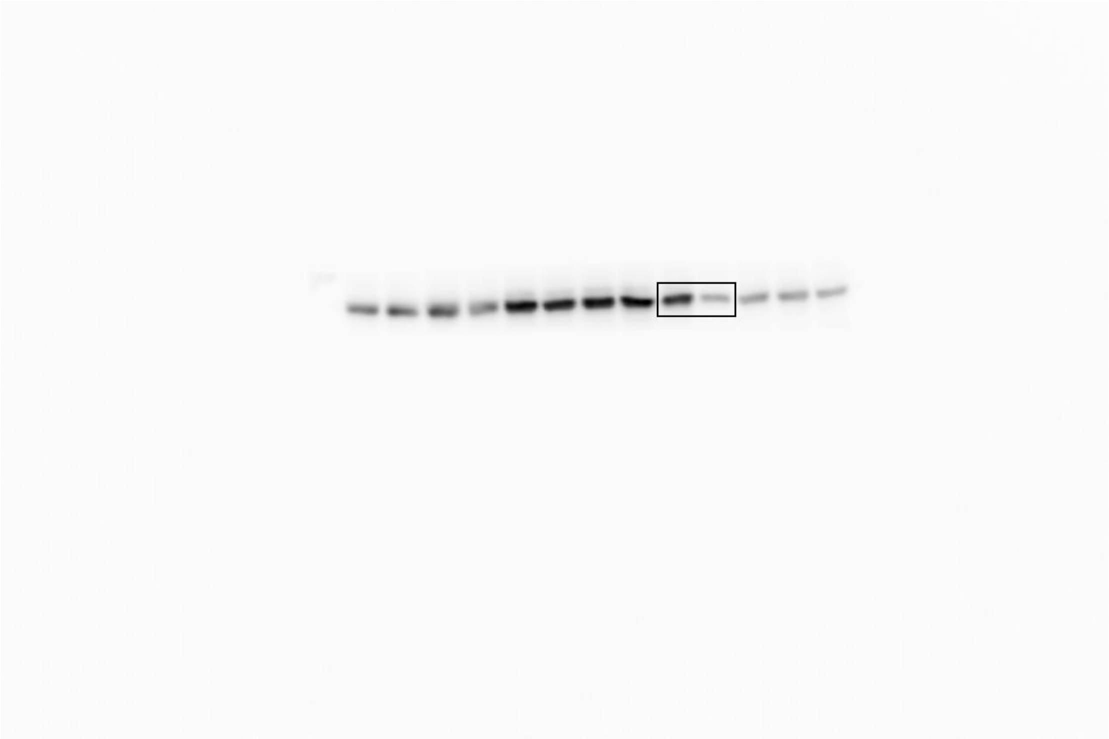

BCKDHA

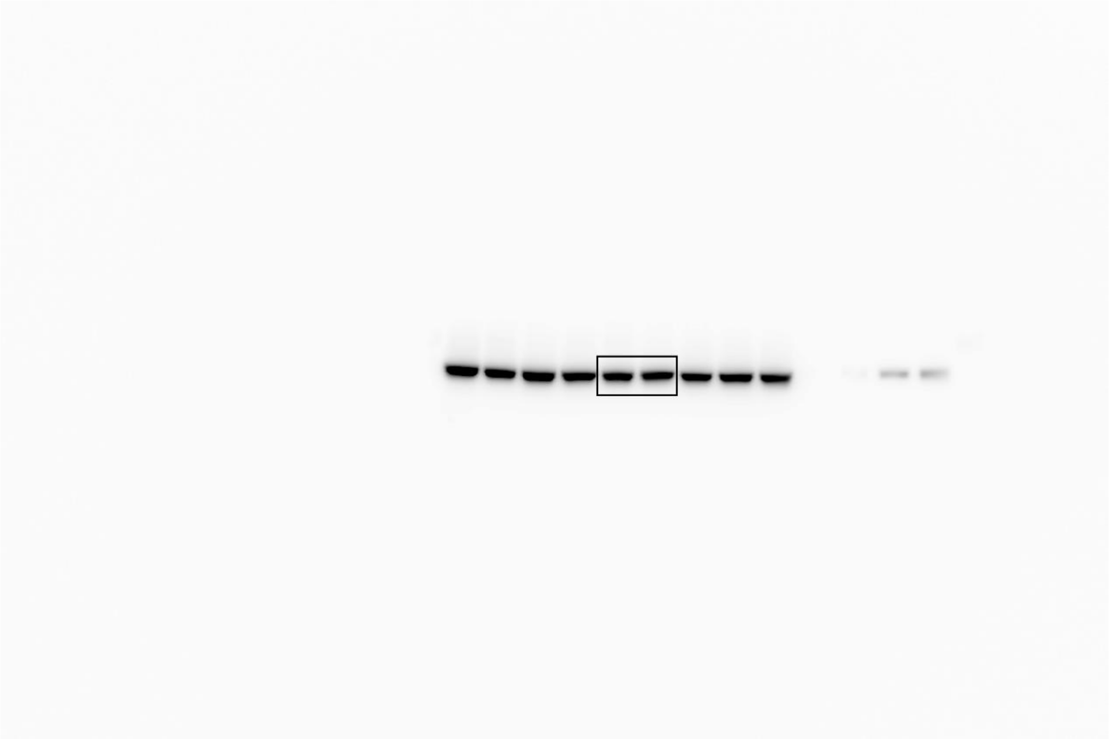

HSP90

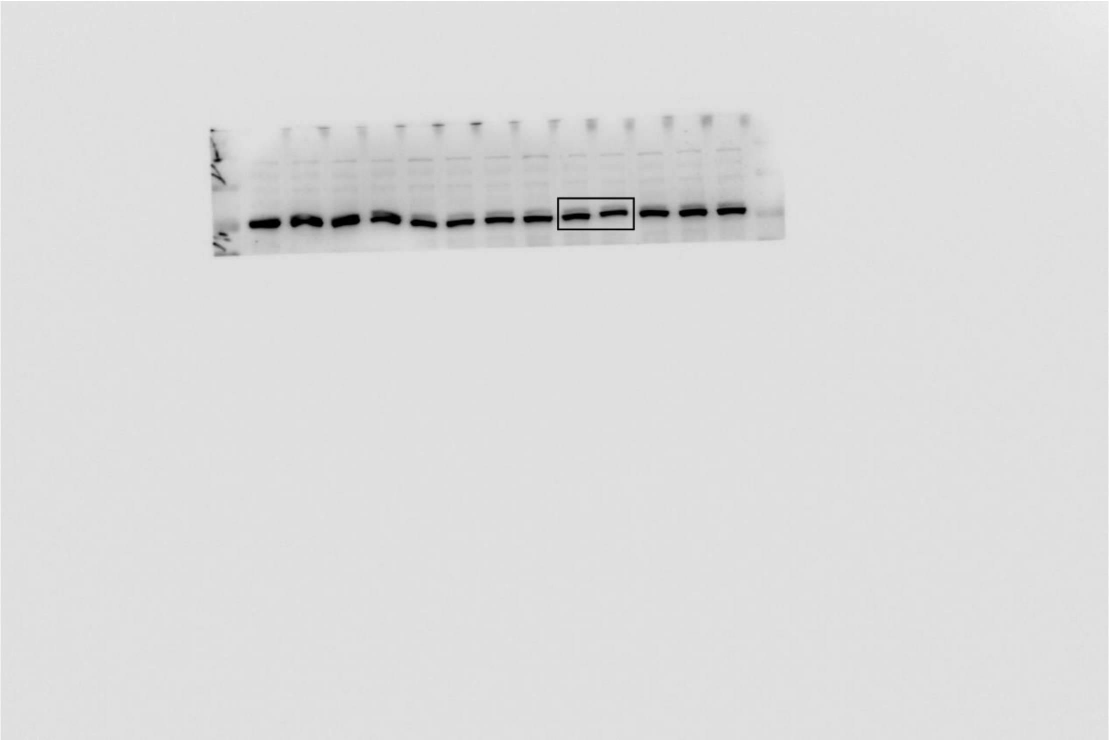

Fig. 4n

PEPCK

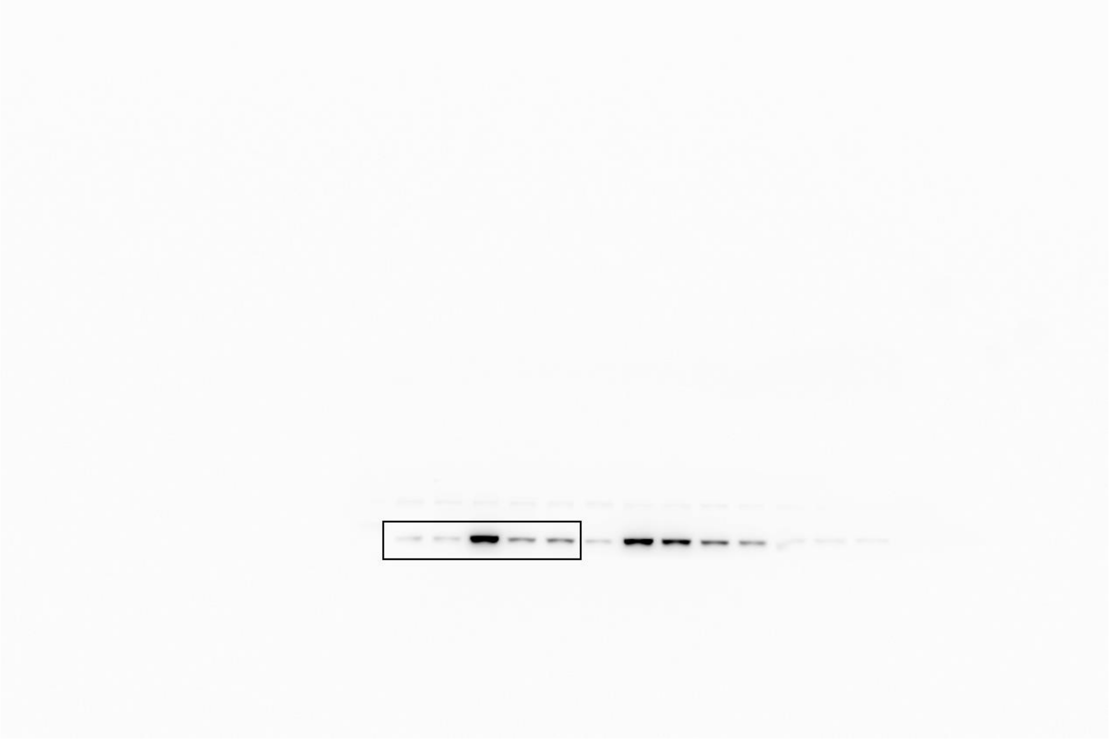

HSP90

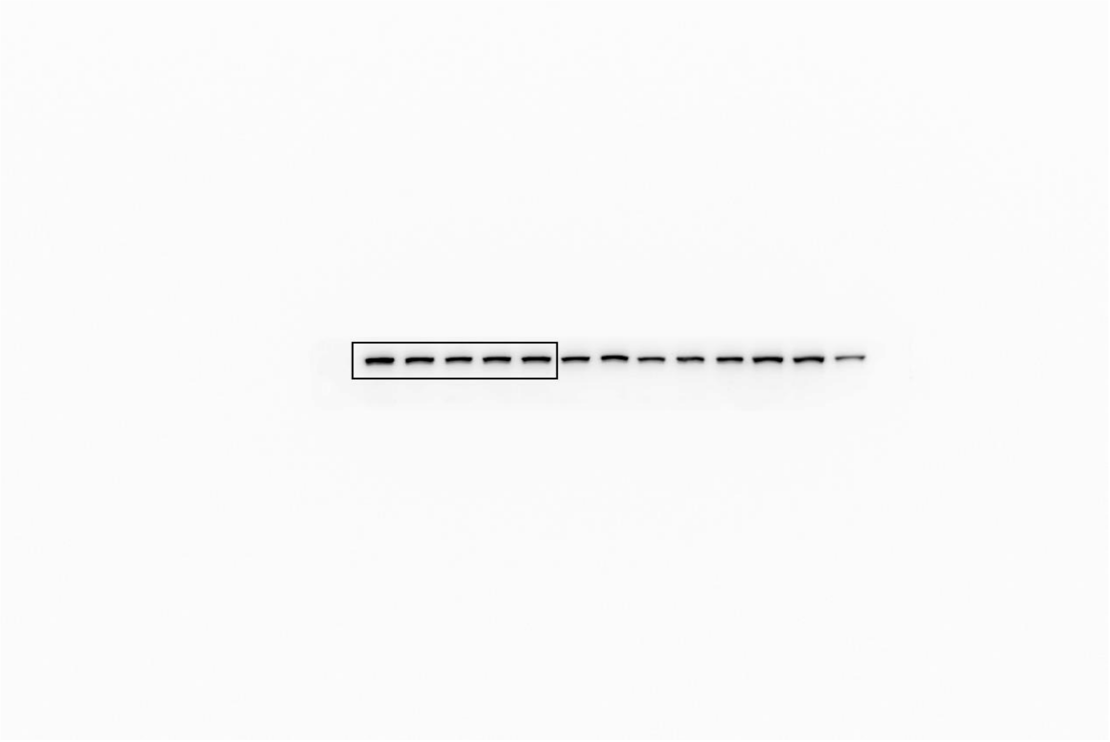

Fig. 5a

FLAG

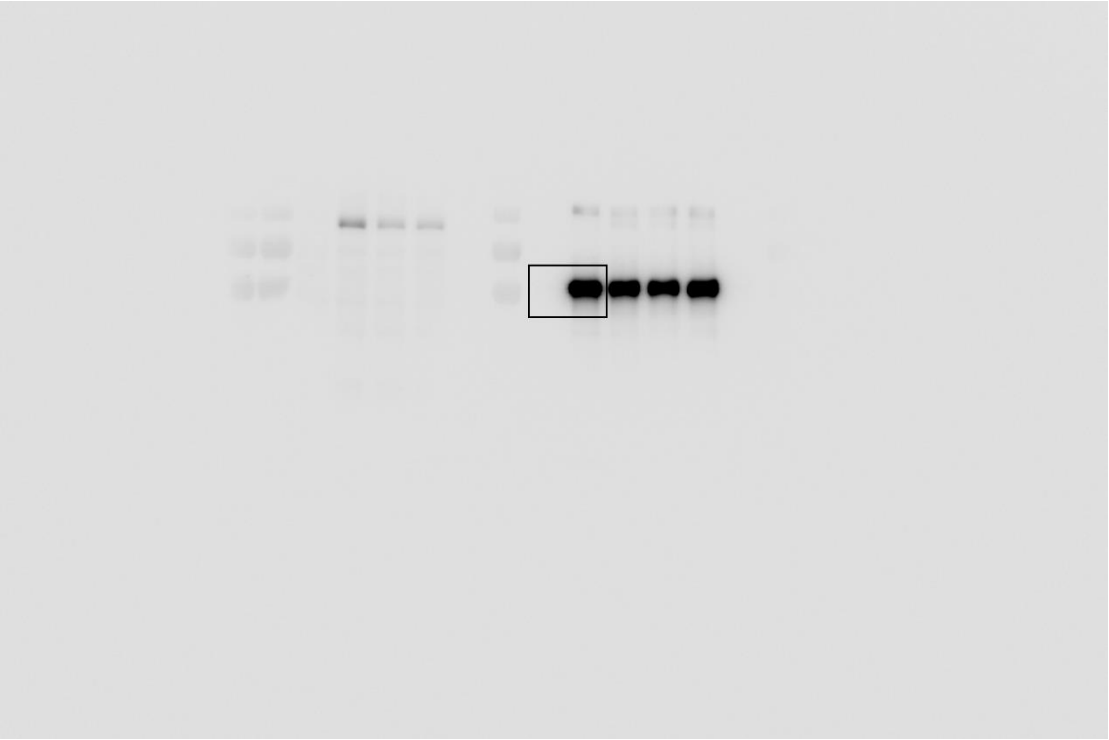

HSP90

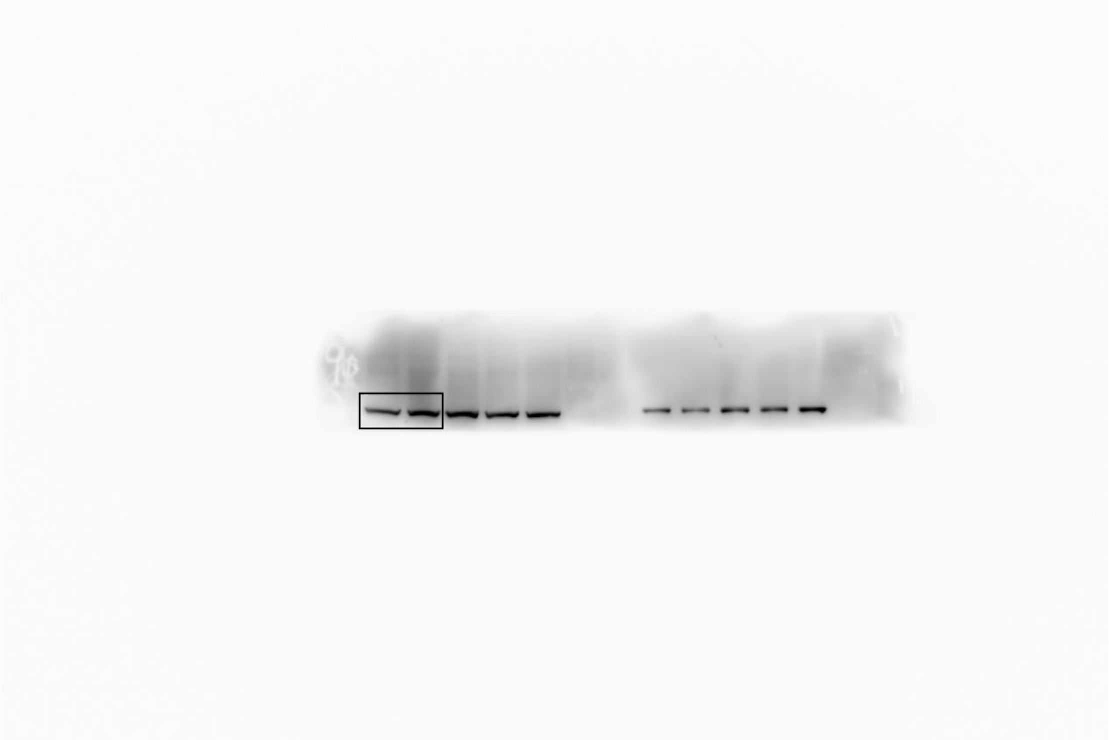

Fig. 7c

P-CREB

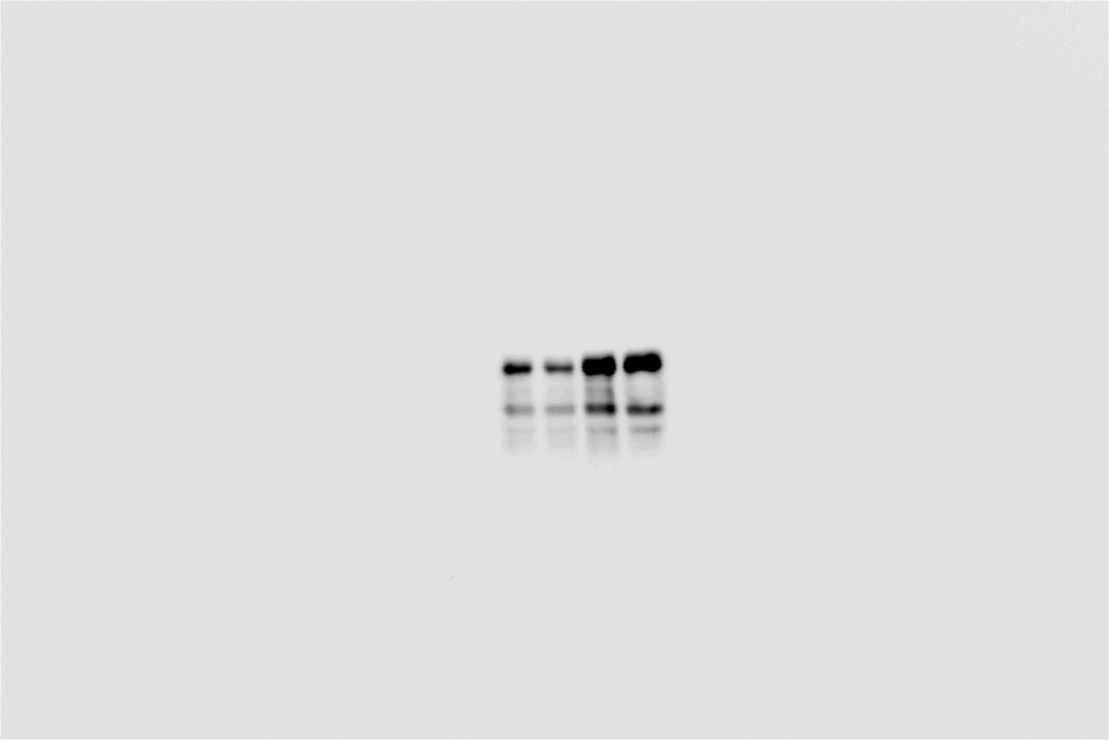

Tubulin

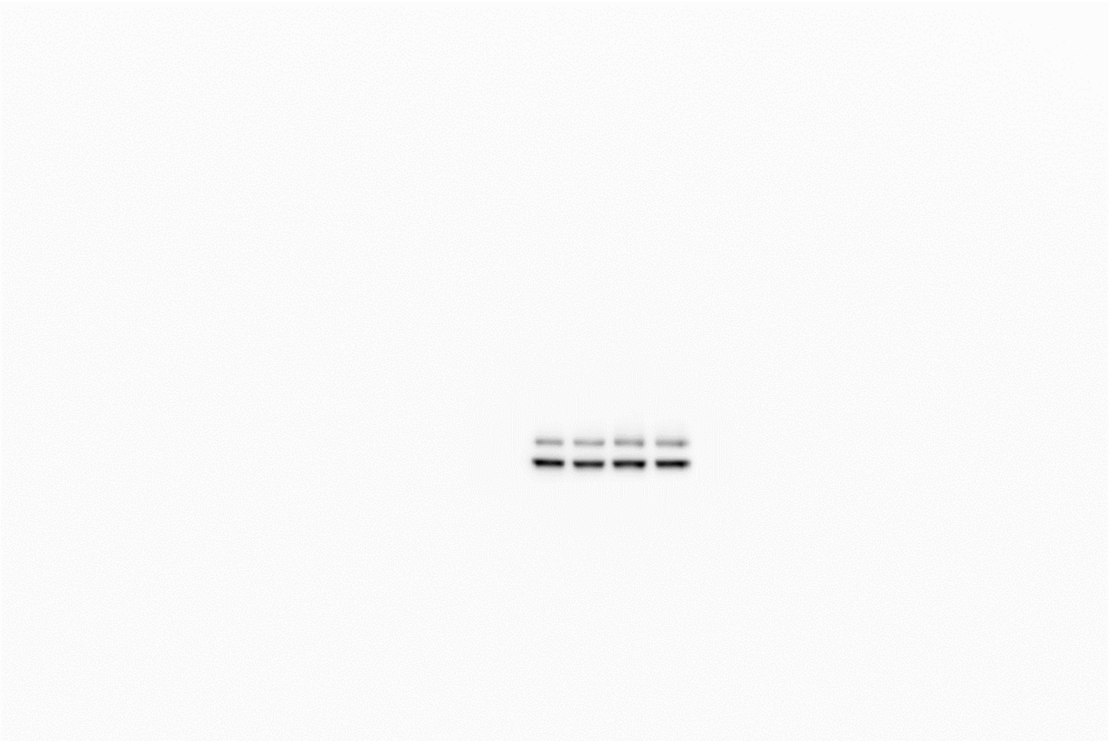

Fig. 7d

CBP

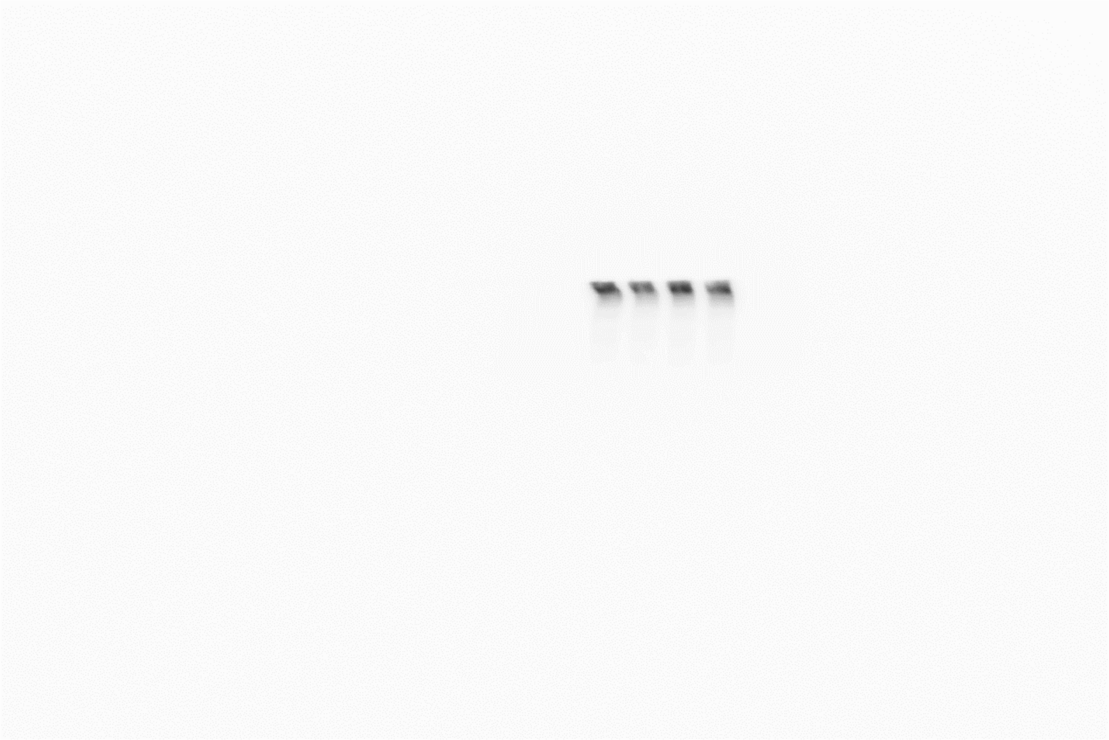

TORC2

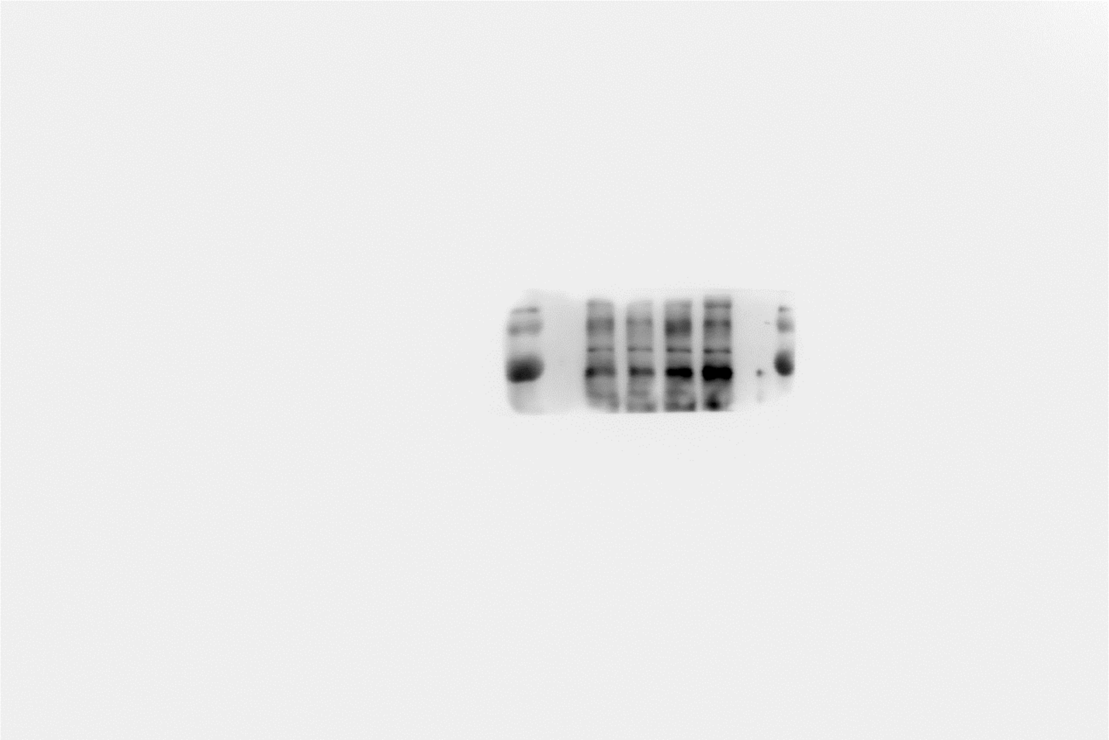

CREB

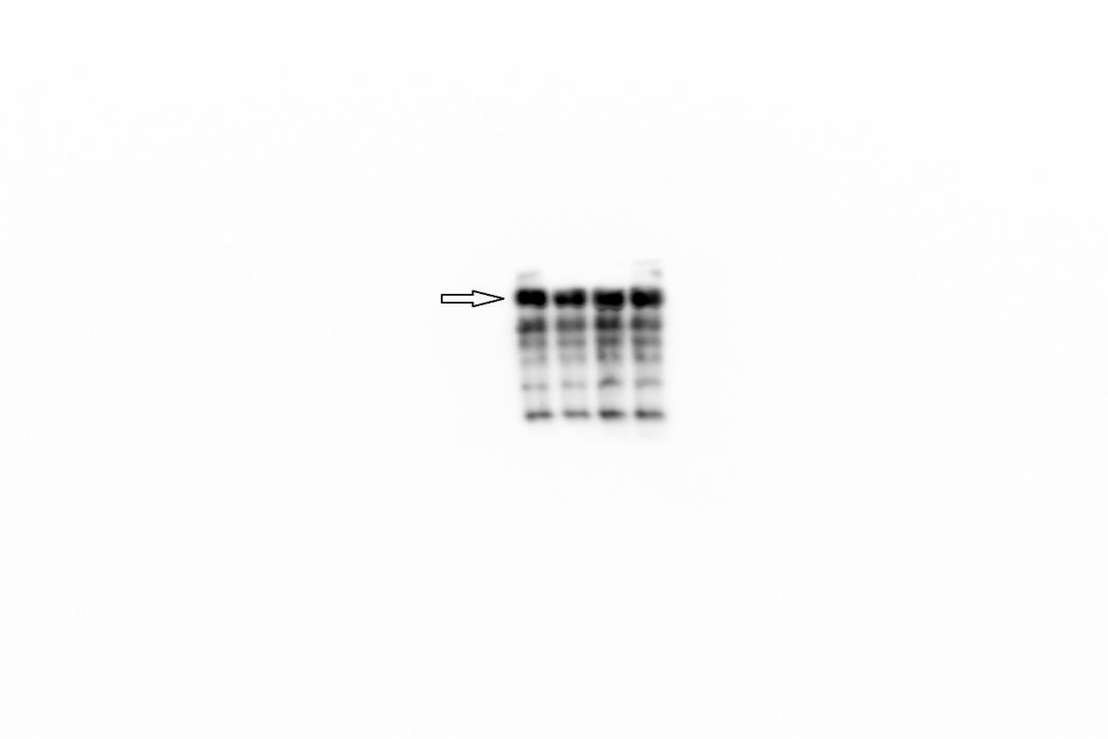

Fig. 7e

CREB

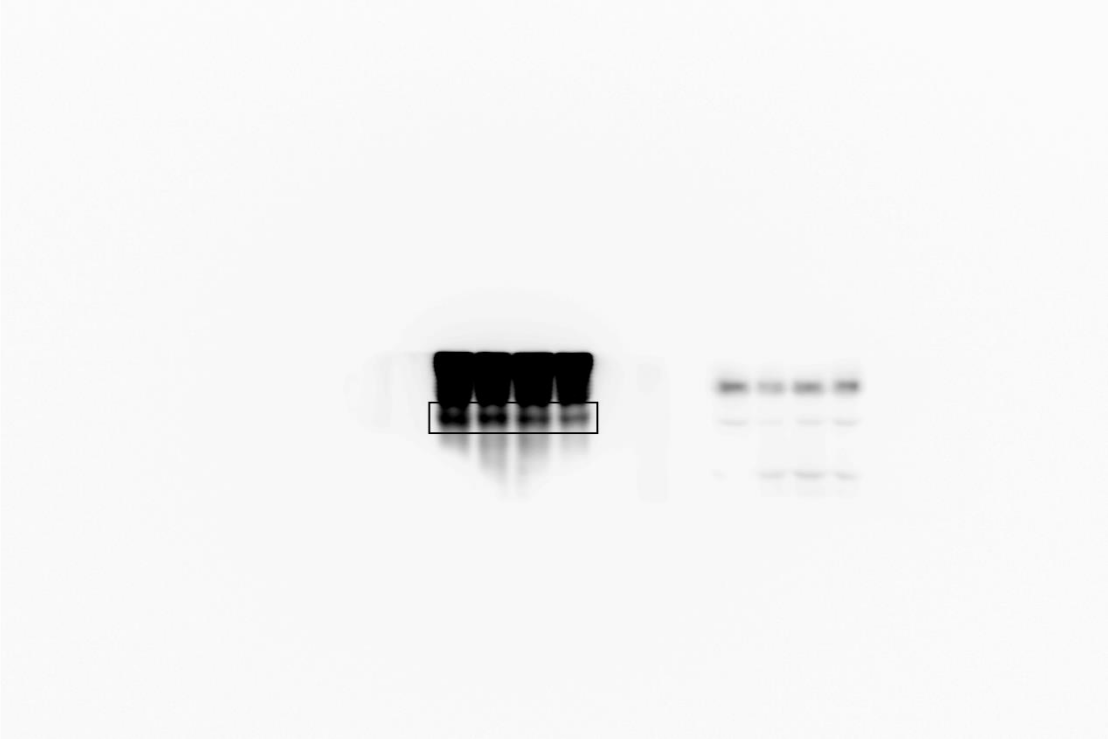

FOXO1

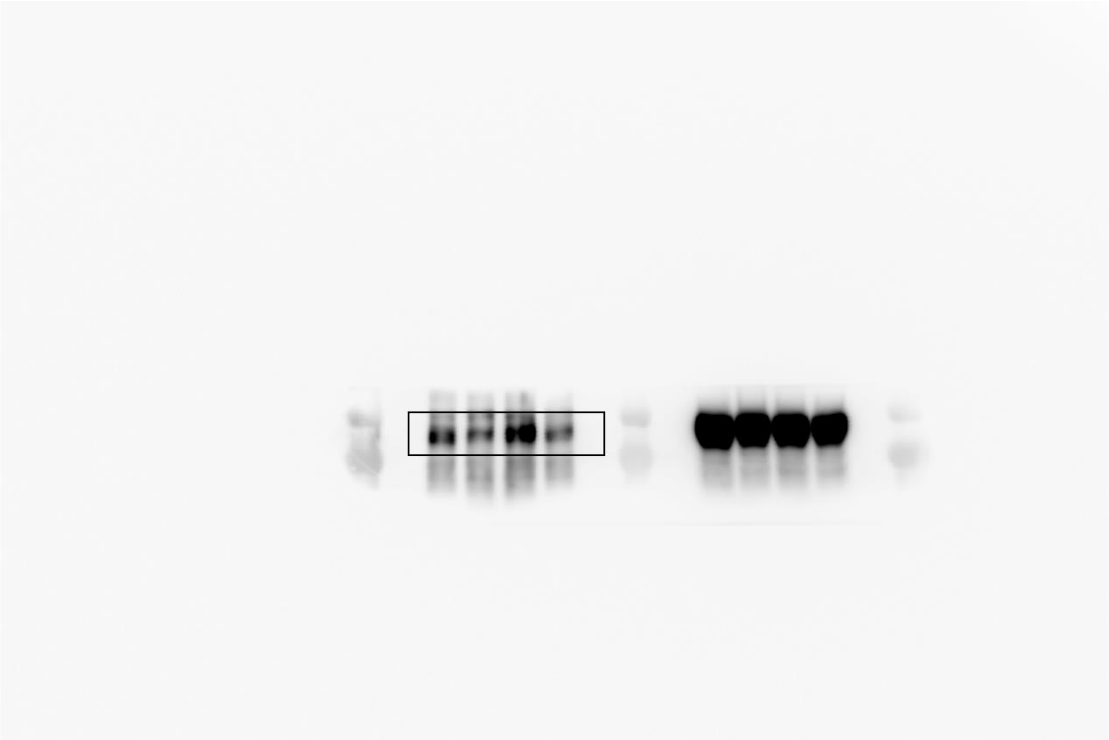

CBP

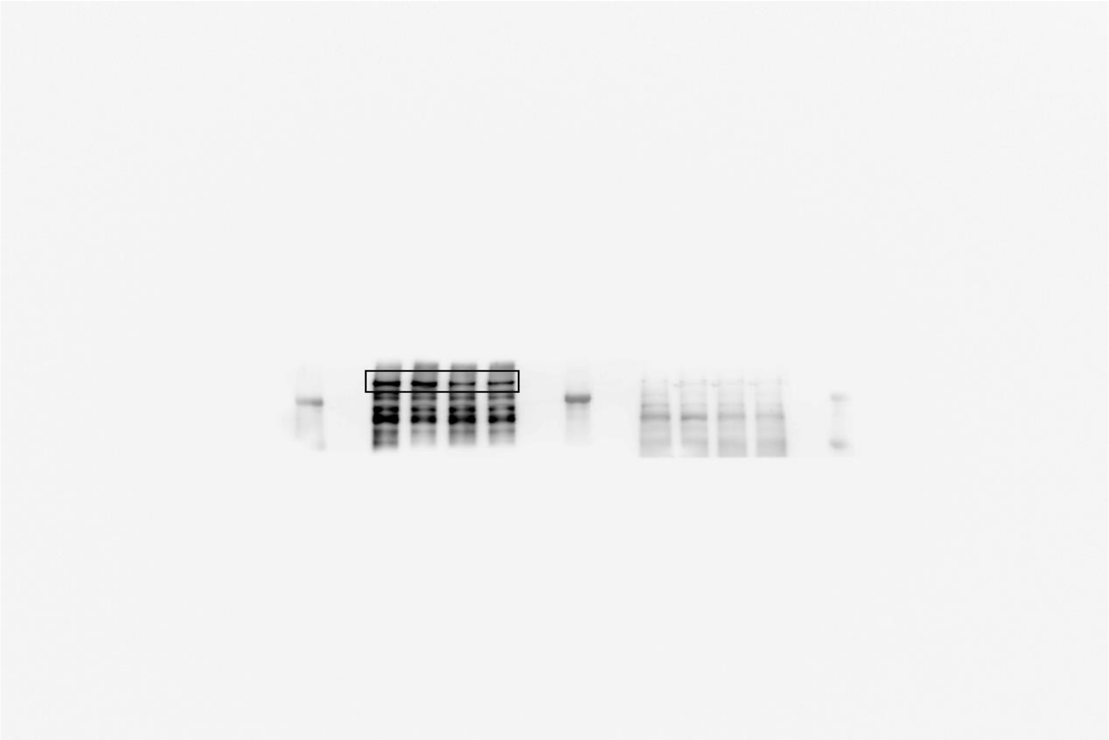

Fig. 7g

FOXO1

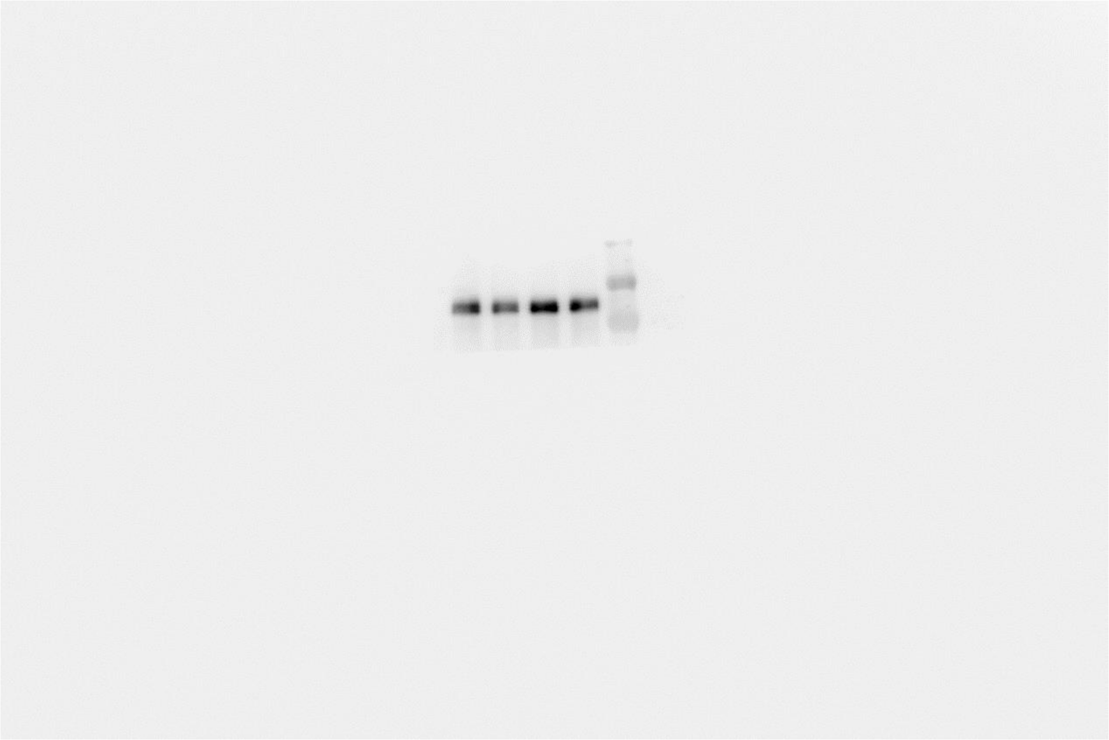

GAPDH

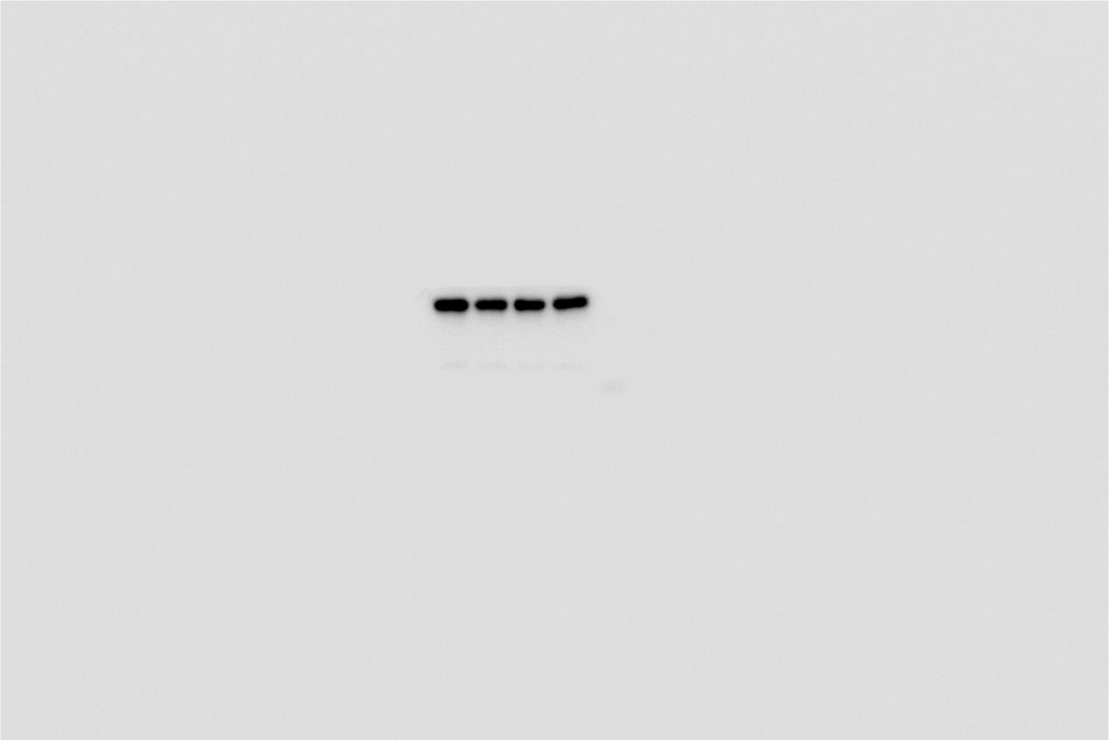

Fig. 7h

FOXO1

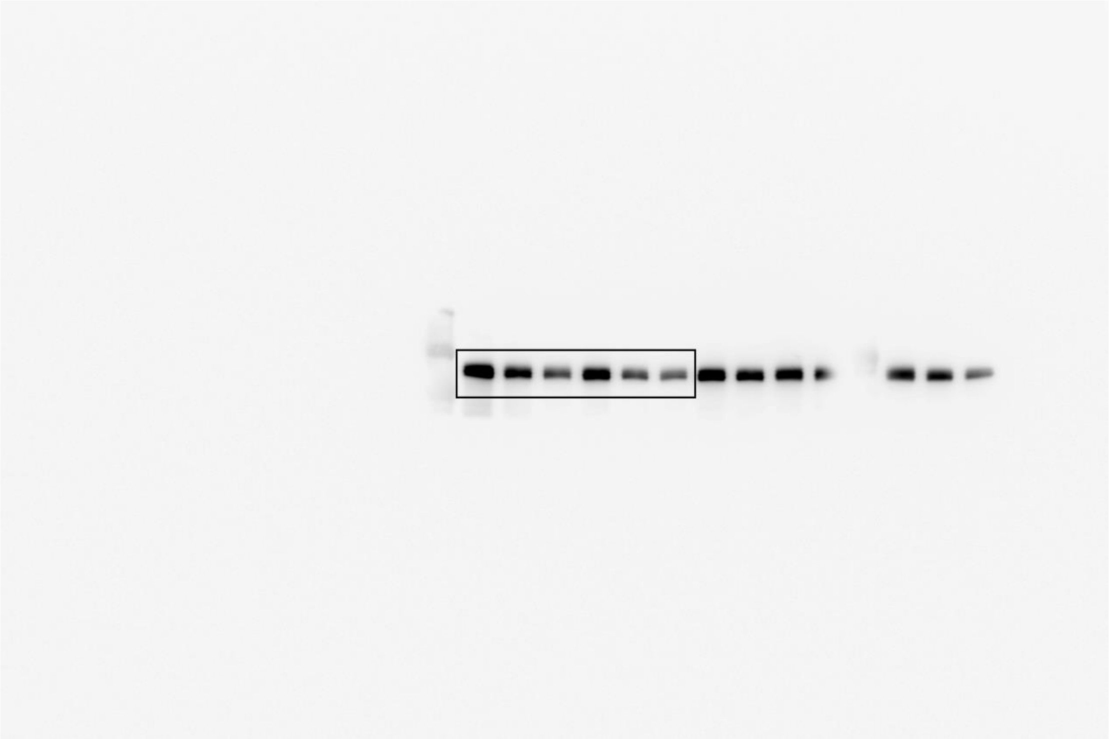

GAPDH

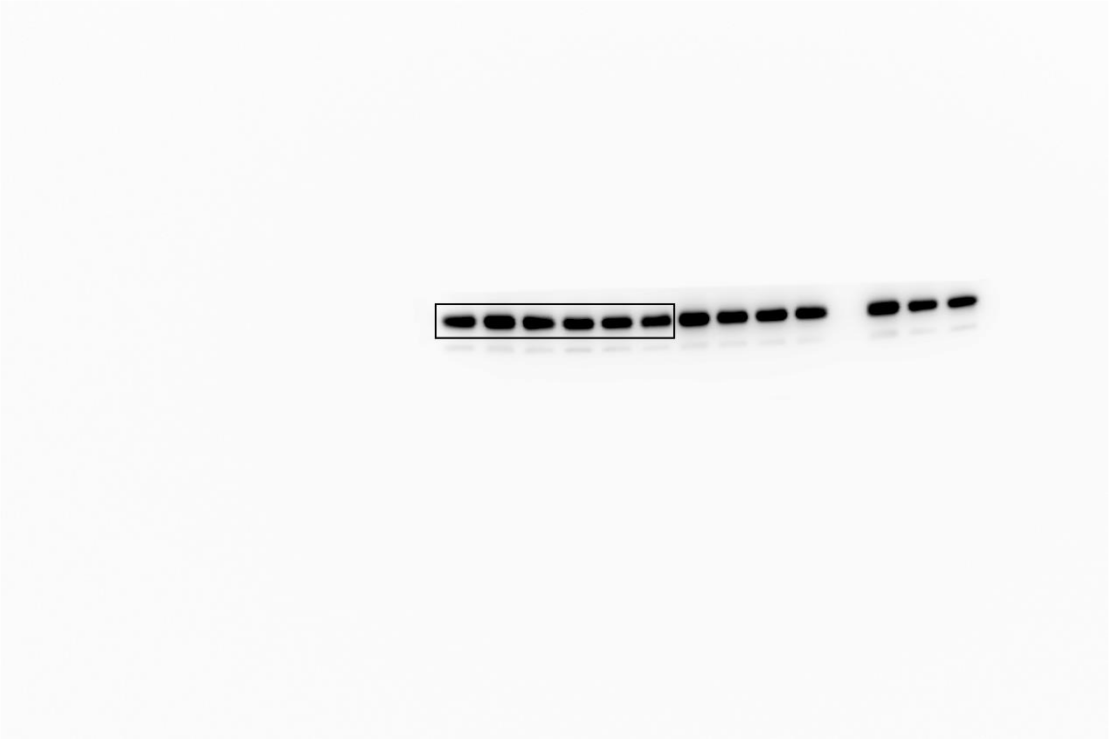

Fig. 7i

FOXO1

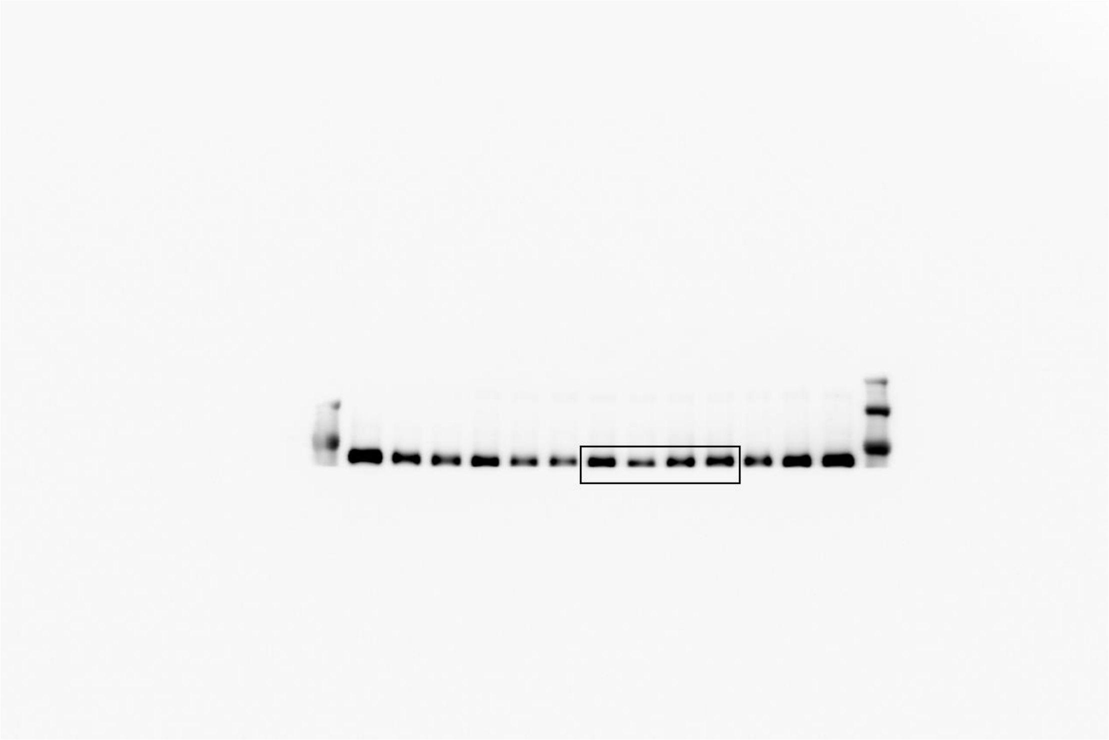

GAPDH

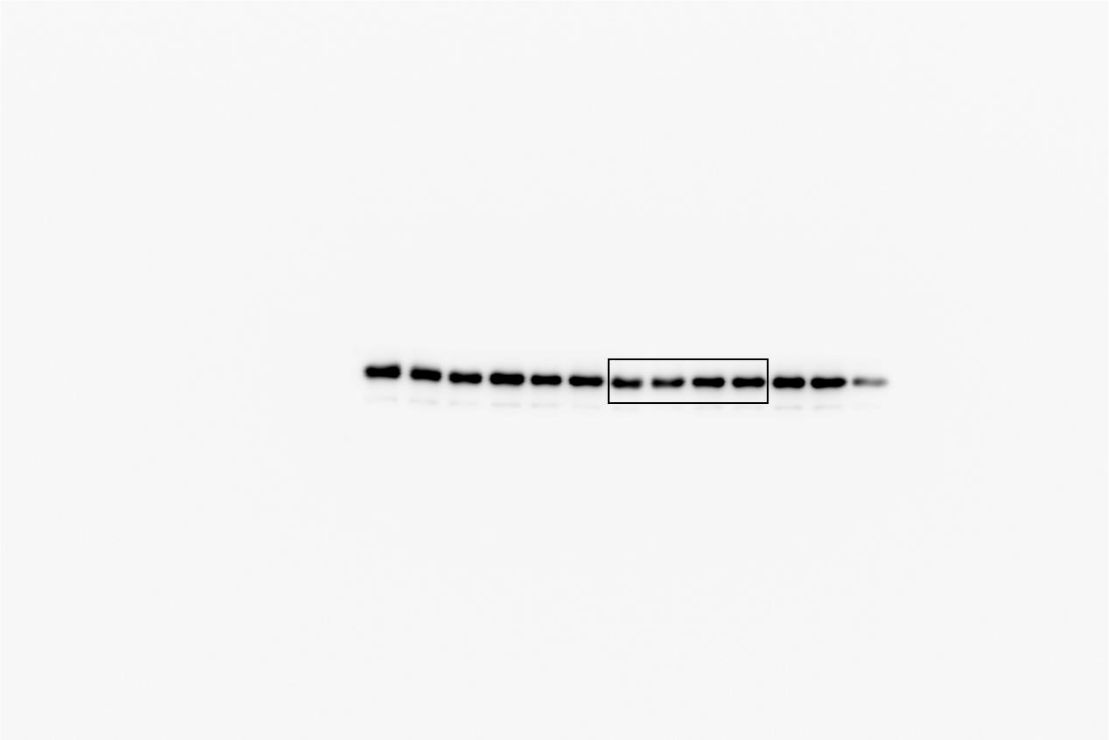

Fig. 7j

HA

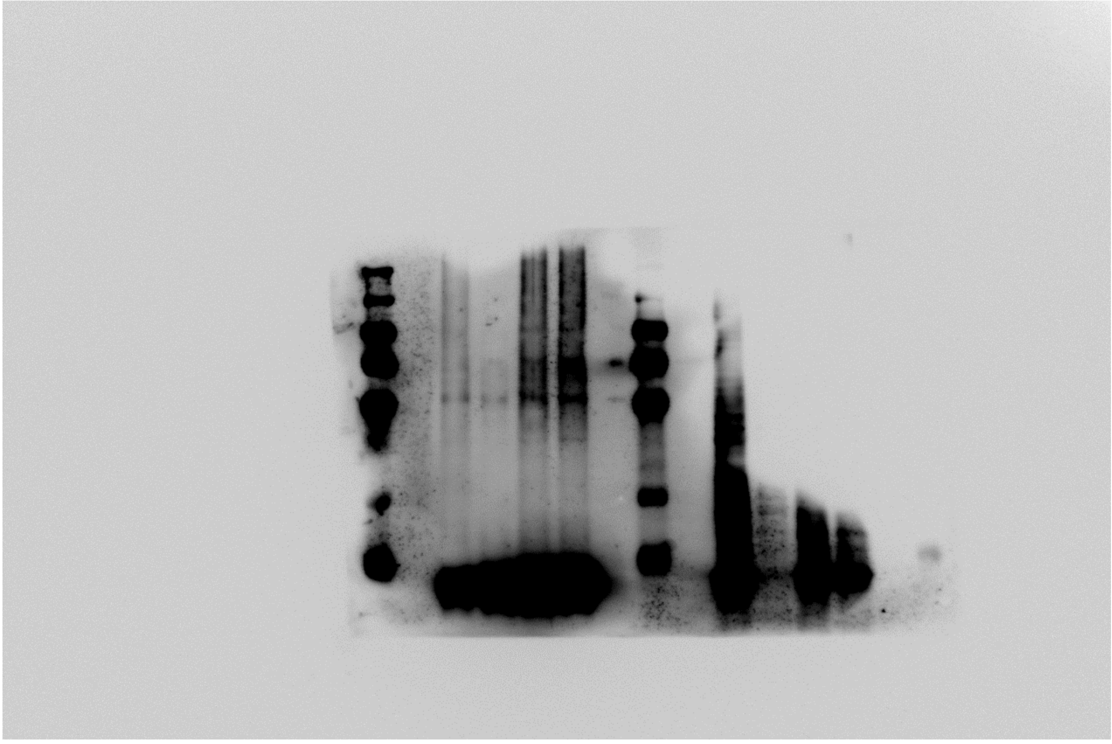

FLAG

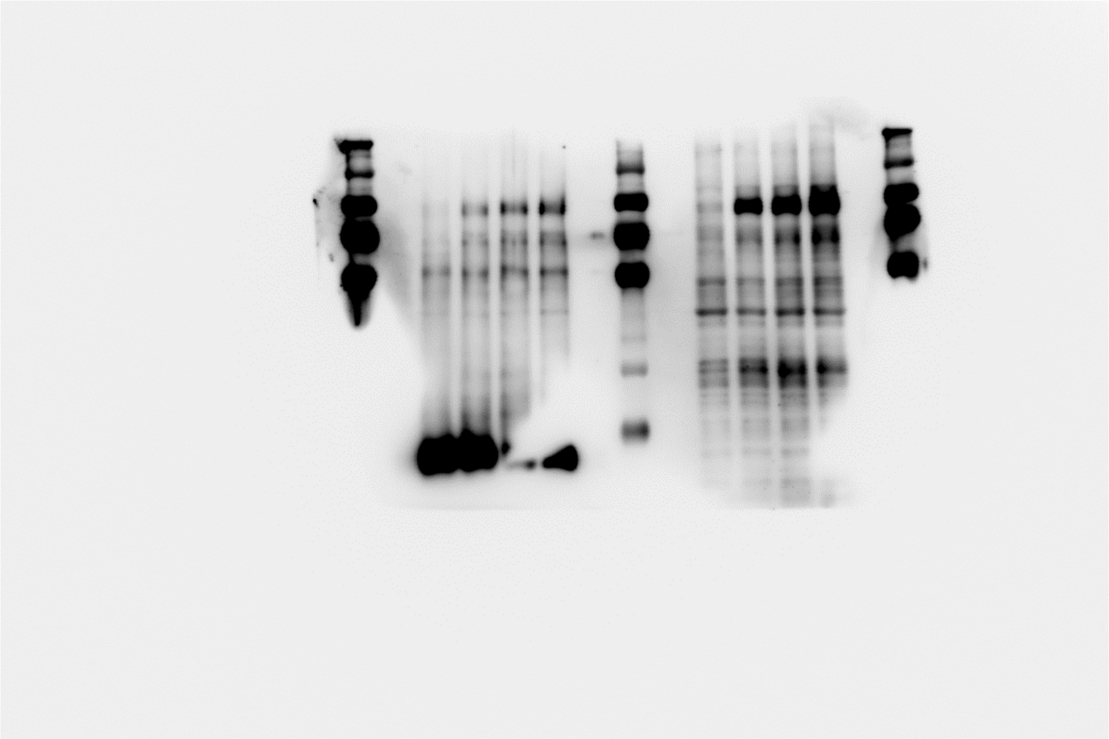

Supplement: Supplementary file 2 — Original full-length western blots [file 41419_2024_7071_MOESM2_ESM.pdf]
